# Quantifying the Dual Effect of Antitumor and Pro-Tumor Human Neutrophils on Natural Killer Cell Behaviors in a Microphysiological System

**DOI:** 10.1021/acsbiomaterials.5c02083

**Published:** 2026-03-18

**Authors:** Shuai Shao, Caroline N. Jones

**Affiliations:** † Department of Bioengineering, 12335 The University of Texas at Dallas, Richardson, Texas 75080, United States; ‡ Department of Biomedical Engineering, UT Southwestern Medical Center, Dallas, Texas 75235, United States

**Keywords:** microphysiological systems, neutrophils, natural killer cells, cancer, migration, cytotoxicity

## Abstract

Neutrophils, the most abundant immune cells in humans, can promote the progression of many solid tumors. Neutrophils in solid tumor tissues can contribute to immunosuppression and resistance to immunotherapy partially by inhibiting the antitumor activity of natural killer (NK) cells, a group of innate immune cells known as the first line of defense against cancer. Studies in mice show that neutrophils are functionally plastic and can be polarized by molecular cues to show either an antitumor “N1” or a pro-tumor “N2” phenotype. However, the crosstalk between neutrophils and NK cells in human cancer is not well characterized, especially as to how different subtypes of neutrophils could influence NK cell behaviors differently. In this study, we engineered a human cell-based microphysiological system to quantify the distinct effects of antitumor N1-like and pro-tumor N2-like neutrophil subtypes on NK cell behaviors including migration and tumor cytotoxicity. We found that NK cells showed preferential migration toward N1-like neutrophils over N2-like neutrophils, although they showed lower motility in terms of speed, displacement, and directionality after migration toward N1-like neutrophils in comparison to N2-like neutrophils. Moreover, N1-like neutrophils restored the NK cell cytotoxicity against pancreatic tumor spheroids, while N2-like neutrophils suppressed it, although both neutrophil subtypes inhibited NK cell infiltration into tumor spheroids. Our study reveals the dual role of human neutrophils in modulating NK cell behaviors and sheds new light on the nuanced crosstalk between different immune cell types, suggesting the reprogramming of neutrophils to enhance the antitumor functions of NK cells as a potential immunotherapy strategy for cancer.

## Graphical abstract

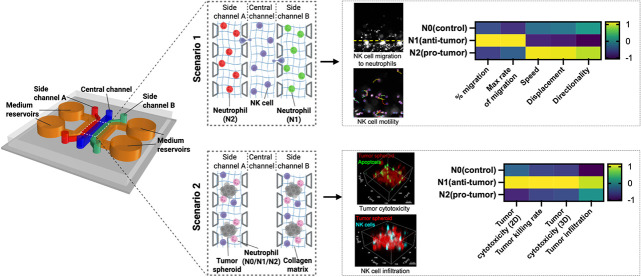

## Introduction

Neutrophils are the most abundant immune cells in humans and the first line of defense against microbial infection.^1–4^ However, neutrophils are a double-edged sword in cancer; they can either inhibit or promote tumor progression.^5^ On the one hand, neutrophils have been widely described to facilitate the growth, angiogenesis, and metastasis of solid tumors, which overturns the primary role of neutrophils in safeguarding the human body against harm.^1,6–13^ On the other hand, studies in mice have demonstrated that inhibition of transforming growth factor-β (TGF-β) promotes the recruitment of cytotoxic, antitumor neutrophils to tumors and curbs tumor growth.^14^ Likewise, additional mouse studies have reported that interferon-β (IFN-β) can polarize neutrophils into an antitumor state and attenuate tumor growth.^15–17^ Collectively, these murine models indicate that neutrophils can be shaped by specific molecular signals to adopt either an antitumor (denoted as “N1”) or a pro-tumor (denoted as “N2”) phenotype.^18–20^ This functional plasticity highlights the therapeutic potential of directing neutrophil polarization toward the antitumor “N1” state.^21^

One way in which neutrophils play a tumor-promoting role is through suppressing the antitumor activity of other immune cells in the tumor microenvironment such as natural killer (NK) cells, a group of innate immune cells known as the first line of defense against cancer.^22–24^ Murine *in vivo* studies^25,26^ and human cell-based *in vitro* studies^13,27^ show that neutrophils can inhibit NK cell migration to and infiltration in tumors and NK cell cytotoxicity against cancer cells. Other studies show that the absence or presence of NK cells controls the antitumor versus pro-tumor state of neutrophils.^28,29^ Hence, the crosstalk between neutrophils and NK cells in human cancer is still controversial and not fully characterized.^22^ Notably, it remains elusive how N1 and N2 human neutrophil subtypes could differentially modulate NK cell behaviors, such as migration and cytotoxicity. It is also unknown whether the N1 polarization of neutrophils could restore or enhance the antitumor activity of NK cells, thus serving as a potential therapeutic strategy. These knowledge gaps arise in part from the difficulty using conventional *in vitro* and *in vivo* models to isolate and quantify interactions between specific cell types within the complex and heterogeneous tumor microenvironment.

Microphysiological systems, including organ-on-a-chip models, have emerged as advanced *in vitro* models for studying immune cell-cancer interactions and assessing cancer immunotherapies.^8,30–200^ By integrating microfluidic technology with 3D cell culture and tissue engineering approaches, these systems more closely mimic critical features of the tumor microenvironment than traditional *in vitro* models, enabling precise spatial control and compartmentalization of distinct cell types.^8,30–32,34,35^ Furthermore, unlike most *in vivo* animal models, microphysiological systems accommodate the use of human cells easily and enable real-time visualization and quantitative analysis of cellular interactions and behaviors at single-cell resolution.^36,37^ Hence, microphysiological systems have been employed to examine neutrophil-cancer interactions^26,27,38–48^ and NK cell-cancer interactions^49–56^ using human cells in recent years. Most of these studies performed end-point measurements, such as cancer cell death. Other studies have used microphysiological systems to quantify the temporal dynamics of NK cell migration in real time.^56–62^ However, none of these studies examined the role of different neutrophil subtypes in NK cell-cancer interactions. A quantitative investigation of the effects of antitumor (N1) and pro-tumor (N2) human neutrophils on NK cell behaviors using both real-time and end-point measurements can provide new insights into the complex role of neutrophils in tumor progression and inform the development of neutrophil-based cancer immunotherapy.

In this study, we engineered a human cell-based microphysiological system termed “NNTI-chip” (NK cell-neutrophil-tumor interactions on-a-chip) to quantify the distinct effects of antitumor N1-like and pro-tumor N2-like neutrophil subtypes on NK cell behaviors including migration, motility, tumor cytotoxicity, and tumor infiltration. We found that NK cells showed preferential migration toward N1-like neutrophils over N2-like neutrophils, although they showed lower motility in terms of speed, displacement, and directionality after migration toward N1-like neutrophils than N2-like neutrophils. Moreover, N1-like neutrophils restored the NK cell cytotoxicity against pancreatic tumor spheroids while N2-like neutrophils suppressed it, although both neutrophil subtypes inhibited NK cell infiltration into tumor spheroids. This study reveals the dual role of human neutrophils in modulating NK cell behaviors and the complex crosstalk between different immune cell types, suggesting reprogramming neutrophils to reverse their immunosuppression on NK cells as a potential immunotherapy strategy for cancer. By serving as a useful tool for both mechanistic studies and preclinical drug evaluation, the NNTI-chip in this study will help to advance the clinical translation of cancer immunotherapies targeting neutrophils.

## Results

### N1-Like Neutrophils Secrete More NK Cell Chemokine IP-10 and Induce Greater NK Cell Activation than N2-Like Neutrophils

In this study, we used the HL-60 human promyelocytic leukemia cell line which is the most commonly used model for primary human neutrophils and has been extensively characterized.^63,64^ We first differentiated HL-60 cells into a neutrophil-like state denoted as dHL-60 neutrophils^63,65^ and then polarized them into either an N1-like (antitumor) or an N2-like (pro-tumor) state as previously described^18,66,67^ (Figure S1A). The unpolarized control was denoted as N0 neutrophils, representing the naïve state.^18,19^ The use of the HL-60 cell line allowed us to achieve stable N1 and N2 polarization *in vitro* for 24 h, without the limitations imposed by the short lifespan (<24 h) and the functional decline of primary human neutrophils after isolation from peripheral blood.^19,48,63^ We validated the identity of our N1-like and N2-like neutrophils by showing that N1-like neutrophils were CD11b^high^/CD54^high^/CD62L^low^/CD182^low^ and N2-like neutrophils were CD11b^low^/CD54^low^/CD62L^high^/CD182^high^ (*p* < 0.0001), which matches the expression patterns of typical N1 and N2 surface markers previously reported^1,18^ (Figure S1B). We found that N1-like neutrophils secrete a significantly higher level of IP-10 (CXCL10), a known chemokine for NK cells,^68,69^ than N2-like and N0 neutrophils (*p* < 0.0001), suggesting a stronger attraction of NK cells toward antitumor (N1) neutrophils (Figure S1C). We also found that N1-like neutrophils secrete higher levels of IFN-γ and TNF-α, which are cytokines known to activate NK cells,^70,71^ than N2-like and N0 neutrophils (Figure S1D). Importantly, the human NK cell line NK-92MI upregulated the degranulation marker CD107a (LAMP-1) (*p* < 0.001) and the activation marker IFN-γ (*p* < 0.0001)^72–74^ upon treatment with conditioned medium from N1-like neutrophils compared to those from N2-like and N0 neutrophils, suggesting the potential of antitumor (N1) neutrophils to boost NK cell cytotoxicity via secretion of soluble factors (Figure S1E).

### NK-92MI Cells Show Preferential Migration toward N1-Like Neutrophils over N2-Like Neutrophils

We developed a microphysiological system “NNTI-chip” to model the preferential migration of NK cells toward different neutrophil subtypes (denoted as “scenario 1”). The NNTI-chip consisted of a central channel (*blue*), side channel A (*red*), side channel B (*green*), and two medium channels, each connected to two medium reservoirs (*orange*) (Figure 1A). The five parallel channels were separated by uniformly spaced trapezoidal microposts that enabled compartmentalization while maintaining interconnectivity. Side channels A and B were seeded with two different subtypes of neutrophils, respectively, to create the following three competitive conditions: N1-like vs N2-like neutrophils, N1-like vs N0 neutrophils, and N2-like vs N0 neutrophils. NK-92MI cells were seeded in the central channel, a setup that enabled us to study their preferential migration toward either neutrophil subtype on the same chip (Figure 1A). NK-92MI cells and neutrophils were separately embedded in a 3D hydrogel made of type I collagen to mimic the extracellular matrix of the tumor tissue. NK-92MI cells were prestained with a labeling dye for tracking their migration, while neutrophils were left unstained for simplicity. The two medium channels were supplied with culture medium to maintain cell viability and minimize evaporation. Time-lapse live imaging was performed to capture NK cell migration toward neutrophils and NK cell motility after migration in real time (Figure 1B,C). We validated that NK-92MI cells maintained their NK cell identity in the NNTI-chip using immunofluorescence of the typical NK cell marker CD56 ^49^ (Figure S2). We also validated using immunofluorescence that N1-like and N2-like neutrophils maintained their polarization states in the NNTI-chip (Figure S3).

**Figure 1:**
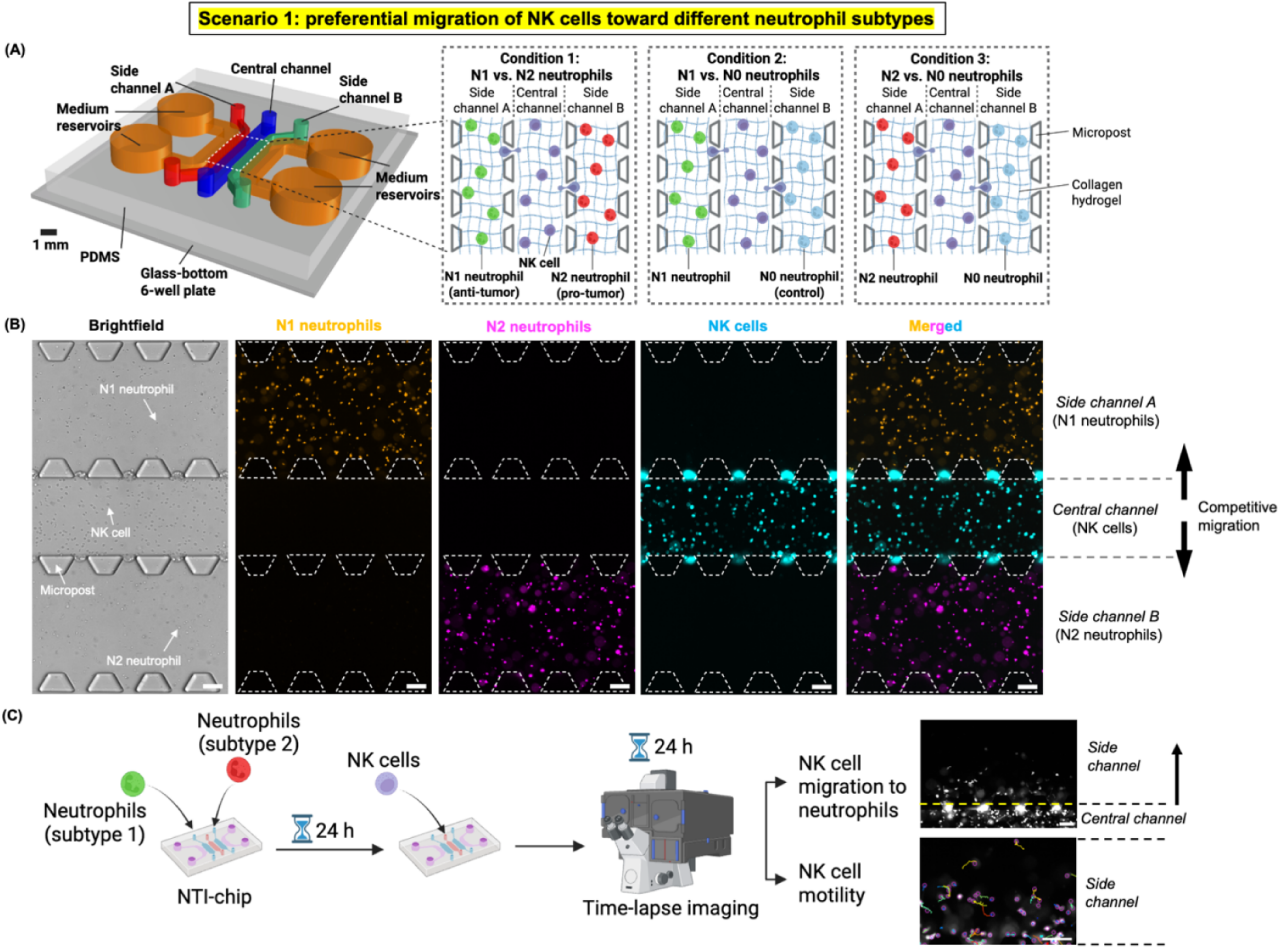
Microphysiological system “NNTI-chip” models the preferential migration of natural killer (NK) cells toward different neutrophil subtypes in scenario 1. (A) Side channels A and B of the NNTI-chip housed two different human neutrophil subtypes (i.e., N1 and N2 neutrophils, N1 and N0 neutrophils, or N2 and N0 neutrophils, respectively) acting as two competitive signals, and NK cells in the central channel were allowed to migrate preferentially into either side channel A or B. Both neutrophils and NK cells were embedded in a 3D collagen hydrogel to mimic the extracellular matrix of the tumor tissue. Created with Rhino 7 and BioRender.com. (B) Representative 10X bright-field and epifluorescence images showing N1 neutrophils (orange) in side channel A, N2 neutrophils (magenta) in side channel B, and NK cells (blue) in the central channel immediately after loading of NK cells into the NNTI-chip. The three channels are separated and interconnected by PDMS microposts. Scale bar, 100 μm. (C) The workflow of a typical experiment for scenario 1. NK cell migration toward neutrophils in both side channels and NK cell motility after migration into side channels were quantified as readouts. Created with BioRender.com.

We first captured the temporal dynamics of NK cell migration from the central channel into side channels A and B housing different neutrophil subtypes every 2 h over 24 h (Figure 2A). NK cell migration to neutrophils was quantified as the percentage of NK-92MI cells that migrated into either side channel A or B. In the N1 vs N2 condition, NK-92MI cells showed a higher percentage of migration to N1-like neutrophils than N2-like neutrophils at all examined time points from *t* = 2 h to *t* = 24 h (12.07% vs 5.11%, *p* < 0.001 at *t* = 24 h) (Figure 2A,Bi; Table S1). In the N1 vs N0 condition, NK-92MI cells showed a higher percentage of migration to N1-like neutrophils than N0 neutrophils at all examined time points from *t* = 2 h to *t* = 24 h (8.60% vs 4.45%, *p* < 0.001 at *t* = 24 h) (Figure 2A,Bi). In the N2 vs N0 condition, there was no significant difference in the percentage of NK-92MI cell migration between N2-like and N0 neutrophils (5.03% vs 5.43%, p= 0.129 at *t* = 24 h) (Figure 2A,Bi). We then calculated the ratio of NK cell migration in side channel B over side channel A at *t* = 24 h for each competitive condition (N1/N2, N1/N0, and N2/N0) and found that the ratios of N1/N2 and N1/N0 were significantly higher than that of N2/N0 (2.52 vs 0.93, *p* < 0.0001; 2.12 vs 0.93, *p* < 0.001) (Figure 2C). We also quantified the maximum rate of NK cell migration, defined as the highest increase in the percentage of migration per hour at any time point for over 24 h (Figure 2Bii). We found that NK-92MI cells showed a higher maximum rate of migration toward N1-like neutrophils than toward N2-like neutrophils (1.31 vs 0.73%/h, *p* < 0.01) and N0 neutrophils (1.31 vs 0.60%/h, *p* < 0.0001) (Figure 2D). Overall, these results showed a clear preference of NK-92MI cells to migrate toward N1-like neutrophils over N2-like neutrophils, suggesting that antitumor (N1) neutrophils may be more capable of attracting and recruiting NK cells than protumor (N2) neutrophils, possibly to fight cancer together. This corroborates our earlier finding that N1-like neutrophils secrete higher levels of the NK cell chemokine IP-10 than N2-like neutrophils (Figure S1C).

**Figure 2:**
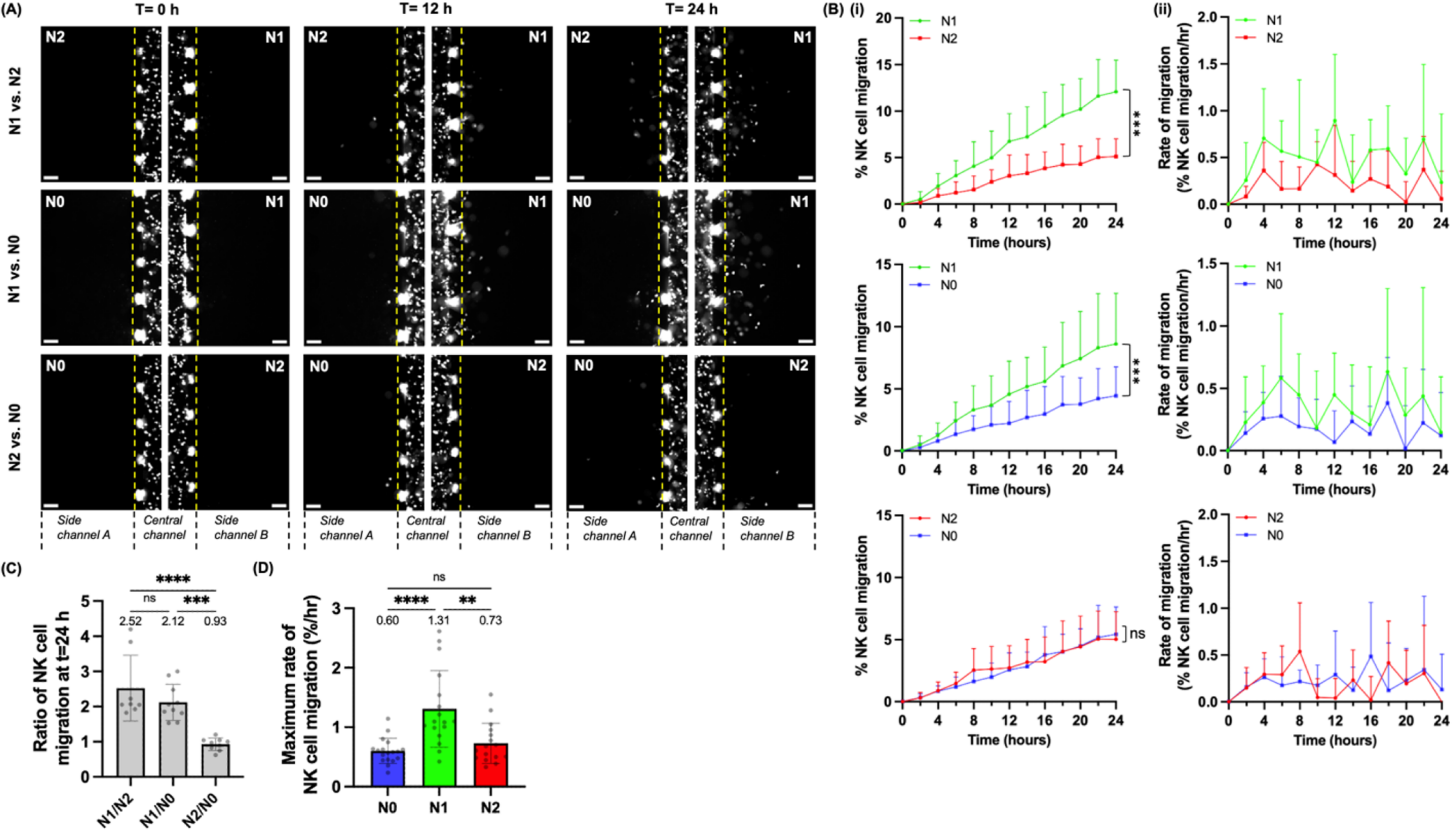
NK-92MI cells show preferential migration toward N1-like neutrophils over N2-like neutrophils. (A) Representative 10X images showing NK-92MI cells (white) migrating from the central channel of the NNTI-chip into side channels A and B under three competitive conditions: N1-like vs N2-like neutrophils, N1-like vs N0 neutrophils, and N2-like vs N0 neutrophils (unstained) at *t* = 0, 12, and 24 h as representative time points. The yellow dashed line marks the boundary between the central channel and the side channels. Scale bar, 100 μm. (B) (i) Line graphs showing the percentage of NK cell migration, defined as the number of NK-92MI cells in side channel A or B at a given time point divided by the initial number of NK-92MI cells in the central channel at *t* = 0 h, every 2 h over 24 h in each competitive condition. Bars show the mean ± SD; *n* = 8–9 chips per condition. Statistical significance is shown for *t* = 24 h. (ii) Line graphs showing the rate of NK cell migration, defined as the increase in the percentage of NK cell migration per hour, every 2 h over 24 h under each competitive condition. Bars show mean ± SD. (C) The ratio of NK cell migration in side channel B over side channel A at *t* = 24 h in three competitive conditions: N1/N2, N1/N0, and N2/N0. Each data point represents an NNTI-chip and *n* = 8–9 chips per condition. (D) The maximum rate of NK cell migration toward N0, N1-like, and N2-like neutrophils, defined as the highest rate of NK cell migration at any time point over 24 h. Each data point represents a side channel and *n* = 16–18 side channels per condition. Bars show mean ± SD with the mean values written above the points. Five independent experiments were performed. ns: *p* > 0.05, *p < 0.05, **p < 0.01, ***p < 0.001, ****p < 0.0001, paired *t* test in (Bi); ANOVA with Tukey multiple comparisons test in (C) and (D).

We also examined NK cell migration in a noncompetitive scenario (“scenario 0”) to validate the results from the competitive scenario (“scenario 1”). Here, side channel A was seeded with N0, N1-like, or N2-like neutrophils in collagen hydrogel or an empty hydrogel control, constituting four different conditions (Figure S4A,B). Side channel B was always seeded with empty hydrogel. The central channel was seeded with NK-92MI cells in collagen hydrogel. The percentage of NK-92MI cell migration to all three neutrophil subtypes was higher than that to the empty gel control over the course of 24 h (*p* < 0.01 for N0; *p* < 0.0001 for N1 and N2), thus showing the ability of NK-92MI cells to migrate toward neutrophils (Figure S4C,D). Importantly, NK-92MI cells showed a higher percentage of migration to N1-like neutrophils than N2-like and N0 neutrophils over the course of 24 h (14.10% vs 8.06%, *p* < 0.001; 14.10% vs 6.63%, *p* < 0.0001 at *t* = 24 h) (Figure S4C,D). We also found that NK-92MI cells showed a higher maximum rate of migration toward N1-like neutrophils than toward N2-like neutrophils (1.40 vs 0.99%/h, *p* < 0.05) and N0 neutrophils (1.40 vs 0.90%/h, *p* < 0.01) (Figure S4E). These results showed the greater migration of NK-92MI cells toward N1-like neutrophils than N2-like neutrophils even in a noncompetitive scenario, validating our earlier findings in the competitive scenario. The reciprocal migration of different neutrophil subtypes toward NK cells was not examined in this study and warrants further investigation.

### NK-92MI Cells Show Lower Motility after Migration toward N1-Like Neutrophils than N2-Like Neutrophils

We also captured the motility of NK-92MI cells after migration into the side channels housing different neutrophil subtypes every 6 h over 24 h of time-lapse imaging in scenario 1. We first extracted the trajectories of individual NK-92MI cells at each time point (*t* = 6, 12, 18, and 24 h) (Figure 3A; Video S1) and then quantified the following motility parameters of each trajectory with single-cell resolution: speed, displacement, and directionality (definitions illustrated in Figure 3B).^63,75,76^ We found that the motility of NK-92MI cells declined over time from *t* = 6 to *t* = 24 h after migration toward both N1-like and N2-like neutrophils in terms of speed and displacement (Figure 3C). Importantly, NK-92MI cells showed an overall higher motility after migration toward N2-like neutrophils than N1-like neutrophils at all examined time points in terms of speed (1.07 vs 0.80 μm/min at *t* = 12 h) and displacement (9.89 vs 6.89 μm at *t* = 12 h) (*p* < 0.0001) (Figure 3D; S5; Table S1). NK-92MI cells also showed a higher directionality after migration toward N2-like neutrophils than N1-like neutrophils at *t* = 24 h (0.43 vs 0.36, *p* < 0.0001) (Figure S5Ciii), although there were no statistically significant differences at earlier time points (*t* = 6, 12, and 18 h) (Figure 3Diii; S5Aiii,Biii). These results showed that N1-like neutrophils reduced the motility of NK-92MI cells compared to N2-like neutrophils, suggesting that antitumor (N1) neutrophils may slow down NK cells after their migration into the tumor tissue compared to pro-tumor (N2) neutrophils. The observed difference may be due to the upregulation of intercellular adhesion molecule ICAM-1 (CD54) by N1-like neutrophils compared to N2-like neutrophils (Figure S1Bii), leading to a stronger adhesion or contact of N1-like neutrophils with NK-92MI cells, which could slow down NK-92MI cells. In addition to direct contact with NK cells, neutrophils could also influence NK cell motility by secreting soluble factors. The molecular mechanisms underlying the differences in NK cell motility between different neutrophil subtypes remain unclear and can be elucidated by measuring the duration of direct contact between neutrophils and NK cells and examining the secretomes of neutrophils in the future. To examine the long-term effect of different neutrophil subtypes on NK cell motility, we can retrieve NK cells from the NNTI-chip after migration to different neutrophil subtypes and measure the spontaneous motility of these differentially exposed NK cells in a future study.

**Figure 3:**
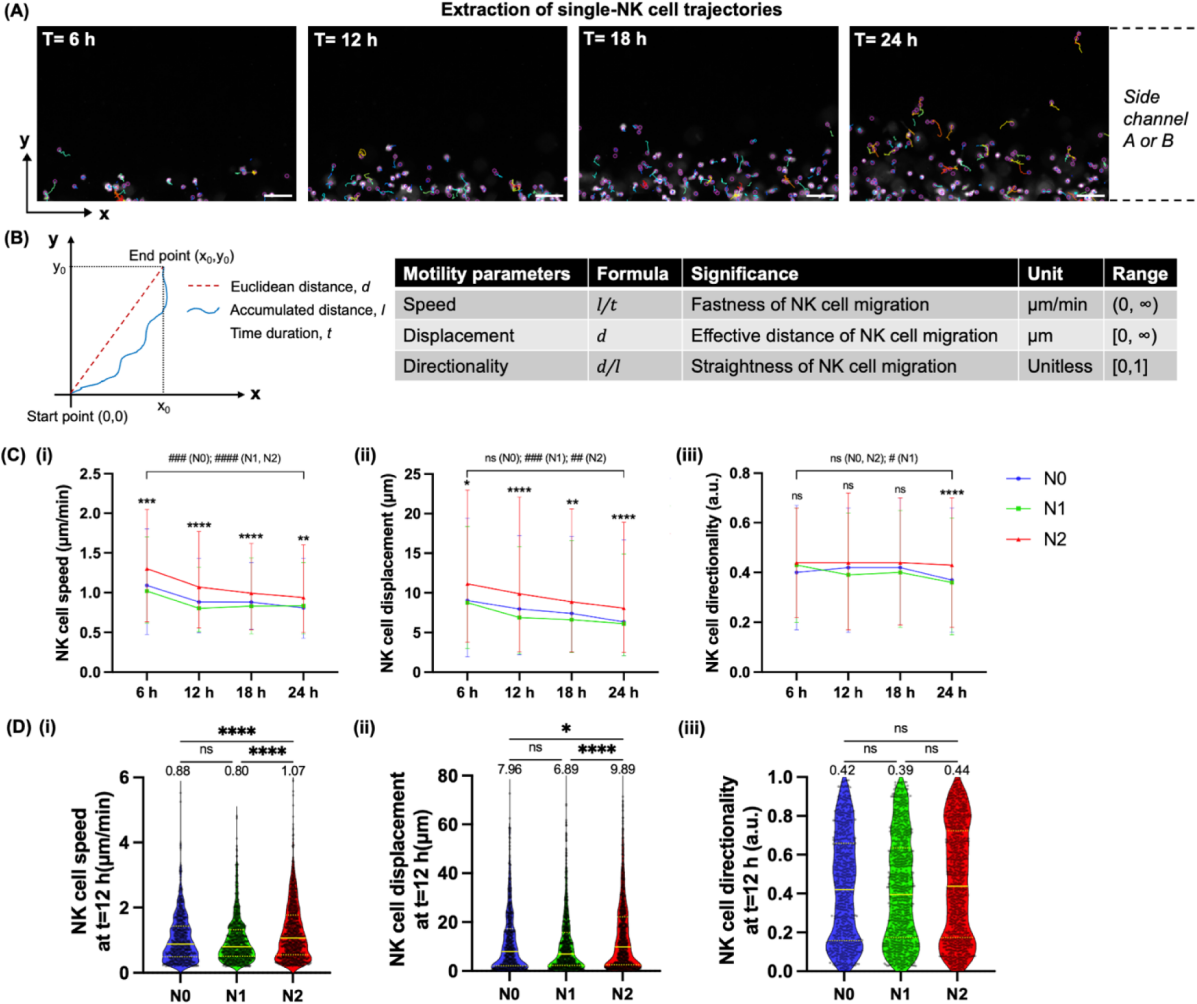
NK-92MI cells show lower motility after migration toward N1-like neutrophils than N2-like neutrophils. (A) Representative 10X images showing the single-cell trajectories (color-coded) of NK-92MI cells (white) after migration into side channel A or B housing neutrophils (not shown) at times of 6, 12, 18, and 24 h of time-lapse imaging. NK-92MI cells were tracked over 30 min intervals every 6 h using TrackMate (ImageJ). Scale bar, 100 μm. (B) Definitions and significance of the following motility parameters of a single-cell trajectory: speed, displacement, and directionality. (C) Line graphs showing the speed (i), displacement (ii), and directionality (iii) of motile NK-92MI cells after migration toward N0, N1-like, and N2-like neutrophils over time at *t* = 6, 12, 18, and 24 h. The median with 25% and 75% percentiles as error bars is shown. *n* = 597–882 cells per condition. Individual data points were not shown for clean data visualization. Asterisks (*) represent the significance of differences between N1 and N2 conditions at each time point, whereas pound signs (#) between *t* = 6 h and *t* = 24 h for each condition (N0, N1, or N2). ns: *p* ≥ 0.05, *p < 0.05, **p < 0.01, ***p < 0.001, ****p < 0.0001; #*p* < 0.05, ##*p* < 0.01, ###*p* < 0.001, ####*p* < 0.0001, Kruskal–Wallis test. (D) Violin plots showing the speed (i), displacement (ii), and directionality (iii) of motile NK-92MI cells with single-cell resolution at *t* = 12 h as a representative time point in specified conditions. Each data point represents a single NK-92MI cell and *n* = 597–882 cells from 17 to 18 side channels of the NNTI-chips per condition. The yellow solid lines show the medians, and the yellow dashed lines show 25% and 75% percentiles. Median values are written above the points. Five independent experiments were performed. ns: *p* ≥ 0.05, *p < 0.05, ****p < 0.0001, Kruskal–Wallis test.

### N1-Like Neutrophils Restore the Cytotoxicity of NK-92MI Cells against PANC-1 Tumor Spheroids, While N2-Like Neutrophils Suppress It

After studying the effect of different neutrophil subtypes on NK cell migration and motility, we used the NNTI-chip to examine the effect of different neutrophil subtypes on NK cell cytotoxicity against cancer, which is another important behavior of NK cells (denoted as “scenario 2”) (Figure 4A). We cultured tumor spheroids which are 3D aggregates of cancer cells from the RFP-PANC-1 human pancreatic cancer cell line (abbreviated as PANC-1) to mimic the 3D shape and structure of solid tumors *in vivo*.^77^ PANC-1 tumor spheroids and NK-92MI cells, together with N0, N1-like, or N2-like neutrophils, were embedded in the same 3D collagen hydrogel which mimics the extracellular matrix of the pancreatic tumor tissue^78–80^ and seeded into side channels A and B of the NNTI-chip (Figure 4A,B). Both side channels of the NNTI-chip were utilized to create the benefit of doubling the throughput or the number of data points collected per experiment.^48^ We characterized the frequency distributions of both the diameter and the number of tumor spheroids loaded per channel. The mean diameter of spheroids was 93.0 ± 18.8 μm (*n* = 873), and each side channel was loaded with an average of 11.3 ± 4.0 spheroids (*n* = 75) (Figure 4C). We also validated using immunofluorescence that N1-like and N2-like neutrophils maintained their polarization states in the presence of tumor spheroids and NK-92MI cells for at least 24 h in the NNTI-chip (Figure S6).

**Figure 4:**
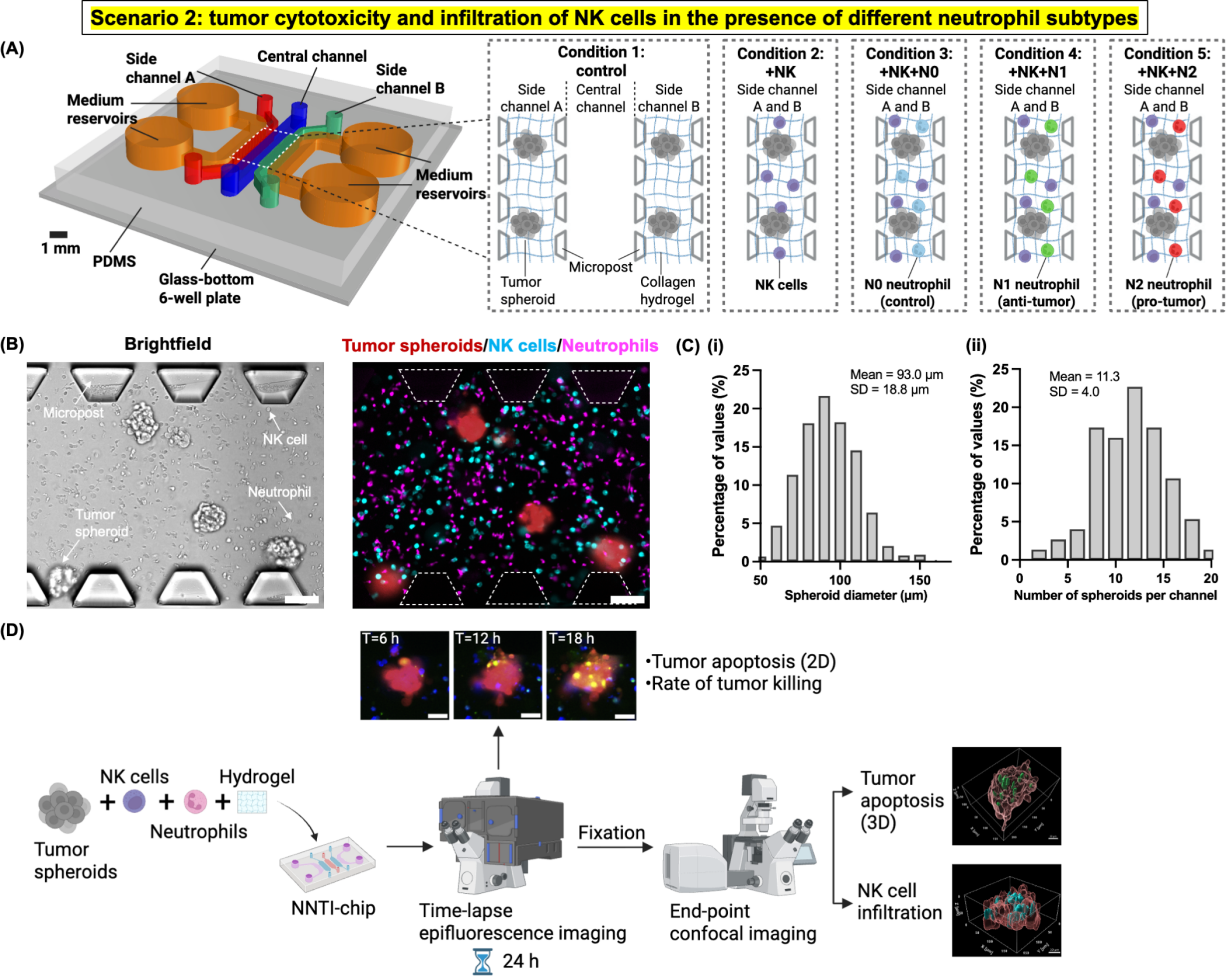
Microphysiological system “NNTI-chip” models the tumor cytotoxicity and infiltration of NK cells in the presence of different neutrophil subtypes in scenario 2. (A) A mixture of tumor spheroids, NK cells, and neutrophils (N0, N1, or N2) was embedded in a 3D collagen hydrogel and seeded in the side channels of the NNTI-chip, which mimics the solid tumor tissue. Side channels A and B served as technical replicates. Created with Rhino 7 and BioRender.com. (B) Representative 10X brightfield and epifluorescence images showing tumor spheroids (red), NK cells (cyan), and neutrophils (magenta) in the same side channel of the NNTI-chip. Scale bar, 100 μm. (C) (i) The frequency distribution of diameters of tumor spheroids loaded into the two side channels of the NNTI-chip. The mean diameter is 93.0 ± 18.8 μm (*n* = 873 spheroids). (ii) The frequency distribution of the number of tumor spheroids loaded into each side channel of the NNTI-chip. The mean number is 11.3 ± 4.0 (*n* = 75 side channels). (D) The workflow of a typical experiment for scenario 2. Real-time tumor spheroid apoptosis in 2D, end-point tumor spheroid apoptosis in 3D, and NK cell infiltration in tumor spheroids were quantified as readouts. Created with BioRender.com.

Using staining of the apoptosis marker caspase-3/7 ^81,82^ and epifluorescence live imaging, we captured the temporal dynamics of tumor spheroid apoptosis every 2 h over 24 h as a measure of NK cell cytotoxicity (Figure 4D; Video S2). Tumor spheroid apoptosis was quantified as the normalized mean fluorescence intensity of caspase-3/7 per spheroid. We first validated that NK-92MI cells were cytotoxic against PANC-1 tumor spheroids, as the apoptosis of tumor spheroids was significantly higher in the presence of NK-92MI cells than alone throughout 24 h (0.88 vs 0.03, *p* < 0.0001 at *t* = 24 h) (Figure 5A,B). We found that the presence of N0 neutrophils reduced tumor spheroid apoptosis induced by NK-92MI cells (0.88 vs 0.61, *p* < 0.01 at *t* = 24 h) (Figure 5A,B), showing that naïve neutrophils can suppress the tumor cytotoxicity of NK cells. Similar to N0 neutrophils, N2-like neutrophils also reduced the NK-92MI cell-induced tumor spheroid apoptosis (0.88 vs 0.51, *p* < 0.0001 at *t* = 24 h) (Figure 5A,B), showing the propensity of the pro-tumor (N2) neutrophil subtype to suppress the tumor cytotoxicity of NK cells. There was no significant difference in tumor spheroid apoptosis between N0 and N2-like neutrophils (0.61 vs 0.51, *p* = 0.575 at *t* = 24 h) (Figure 5B), suggesting that naïve neutrophils may resemble the pro-tumor phenotype even without N2 polarization. Importantly, N1-like neutrophils increased the NK-92MI cell-induced tumor spheroid apoptosis compared to N0 neutrophils (0.61 vs 0.91, *p* < 0.001) and N2-like neutrophils (0.51 vs 0.91, *p* < 0.0001) and restored tumor apoptosis to a similar level as the condition of NK-92MI cells only without neutrophils (0.91 vs 0.88, *p* > 0.999 at *t* = 24 h) (Figure 5A,B; Table S1). This suggests that the antitumor (N1) neutrophil subtype can rescue the tumor cytotoxicity of NK cells impaired by the naïve (N0) and pro-tumor (N2) subtypes. In addition, we found that N0, N1-like, or N2-like neutrophils did not affect tumor spheroid apoptosis in the absence of NK-92MI cells (control vs N0 vs N1 vs N2: 0.04 vs 0.04 vs 0.04 vs 0.03, *p* > 0.999 at *t* = 24 h) (Figure S7). This means that the tumor spheroid apoptosis observed in the tricoculture conditions was mediated by NK-92MI cells rather than neutrophils.

**Figure 5:**
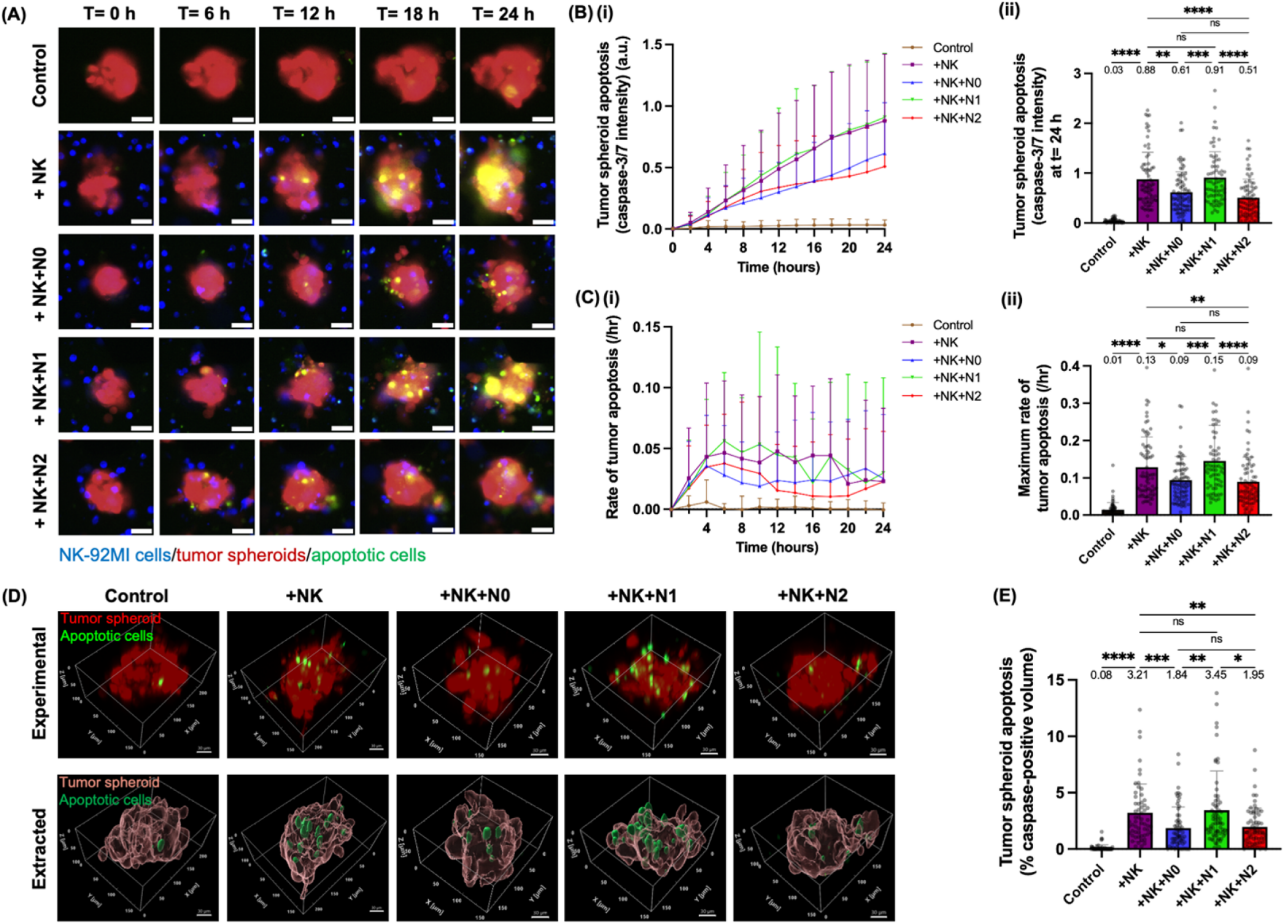
N1-like neutrophils restore the cytotoxicity of NK-92MI cells against PANC-1 tumor spheroids, while N2-like neutrophils suppress it. (A) Representative 10X epifluorescence images showing the apoptosis (caspase-3/7 green) of tumor spheroids (red) in the NNTI-chip at 0, 6, 12, 18, and 24 h as representative time points in the following conditions: alone (control), with NK-92MI cells (blue), with NK-92MI cells and N0, N1-like, or N2-like neutrophils (unstained). Scale bar, 50 μm. (B) (i) Line graph showing the temporal dynamics of tumor spheroid apoptosis, quantified as normalized caspase-3/7 green intensity per spheroid, every 2 h over 24 h in all five conditions. Bars show mean ± SD. (ii) Bar plot showing tumor spheroid apoptosis at *t* = 24 h. Bars show mean ± SD with mean values written above the points. Each data point represents a tumor spheroid and *n* = 68–83 spheroids per condition. (C) (i) Line graph showing the rate of tumor apoptosis, defined as the increase in tumor spheroid apoptosis per hour, every 2 h over 24 h in all five conditions. Bars show mean ± SD. (ii) Bar plot showing the maximum rate of tumor apoptosis, defined as the highest rate of tumor apoptosis at any time point over 24 h. Bars show mean ± SD. (D) 3D rendering of representative 10X confocal images showing the apoptosis (caspase-3/7 green) of tumor spheroids (red) fixed at *t* = 24 h and the extraction of the apoptotic volume and the total spheroid volume by Imaris. Images were acquired as z-stacks with a step size of 2 μm. Apoptotic signals colocalized with DiD-stained NK-92MI cells were excluded to remove apoptotic NK cells. Tumor apoptosis was quantified as the apoptotic volume divided by the total spheroid volume. Scale bar: 30 μm. (E) Bar plot showing the percentage of apoptotic volume per tumor spheroid. Each data point represents a spheroid and *n* = 58–72 spheroids per condition. Bars show mean ± SD with mean values written above the points. At least three independent experiments were performed. ns: *p* ≥ 0.05, *p < 0.05, **p < 0.01, ***p < 0.001, ****p < 0.0001, Kruskal–Wallis test.

Based on the temporal information on tumor spheroid apoptosis over 24 h, we calculated the rate of tumor apoptosis, defined as the increase in tumor spheroid apoptosis per hour (Figure 5Ci). We then quantified the maximum rate of tumor apoptosis, defined as the highest rate of tumor apoptosis at any time point over 24 h, as a measure of the tumor-killing rate of NK cells (Figure 5Cii). We found that both N0 and N2-like neutrophils reduced the maximum rate of tumor apoptosis induced by NK-92MI cells (0.13 vs 0.09/h, *p* = 0.035 for N0; 0.13 vs 0.09/h, *p* < 0.01 for N2) (Figure 5Cii). N1-like neutrophils led to a higher maximum rate of tumor apoptosis induced by NK-92MI cells than N0 neutrophils (0.15 vs 0.09/h, *p* < 0.001) and N2-like neutrophils (0.15 vs 0.09/h, *p* < 0.0001) (Figure 5Cii; Table S1). The presence of N1-like neutrophils also helped NK-92MI cells maintain the same level of tumor-killing rate (0.15 vs 0.13/h, *p* > 0.999) (Figure 5Cii).

We also performed end-point z-stack confocal imaging of the NNTI-chip fixed at *t* = 24 h in a 3D format, which gives a more precise measurement of tumor spheroid apoptosis than epifluorescence imaging in a 2D format (Figure 4D). We measured the total volume and the caspase-3/7-positive apoptotic volume of each tumor spheroid and quantified apoptosis as the percentage of apoptotic volume per spheroid (Figure 5D,E). Similarly, both N0 and N2-like neutrophils reduced 3D tumor apoptosis induced by NK-92MI cells (3.21% vs 1.84%, *p* < 0.001 for N0; 3.21% vs 1.95%, *p* < 0.01 for N2) (Figure 5E). N1-like neutrophils led to a higher 3D tumor apoptosis induced by NK-92MI cells than N0 neutrophils (3.45% vs 1.84%, *p* < 0.01) and N2-like neutrophils (3.45% vs 1.95%, *p* = 0.016) (Figure 5E). The presence of N1-like neutrophils also helped NK-92MI cells maintain the same level of 3D tumor cytotoxicity (3.45% vs 3.21%, *p* > 0.999) (Figure 5E). These results validated the earlier findings based on 2D epifluorescence time-lapse images (Figure 5B). Overall, these results suggest that antitumor (N1) neutrophils can restore the tumor cytotoxicity of NK cells and reverse the immunosuppression mediated by naïve (N0) and pro-tumor (N2) neutrophils. As ICAM-1 is known to be an important costimulatory signal for NK cell activation,^83^ the boost in NK cell cytotoxicity by N1-like neutrophils could be partially explained by the upregulation of ICAM-1 on the surface of N1-like neutrophils compared to N2-like neutrophils (Figure S1Bii), and thus NK-92MI cells may experience further activation while interacting with the ICAM-1 molecules on N1-like neutrophils. The on-chip results also corroborate our well plate-based finding that conditioned medium from N1-like neutrophils resulted in higher expression levels of the degranulation marker CD107a and the activation marker IFN-γ by NK-92MI cells than those from N0 and N2-like neutrophils (Figure S1D). It remains unclear what N1-like neutrophil-derived soluble factors are responsible for maintaining NK cell cytotoxicity and what N0 and N2-like neutrophil-derived soluble factors are responsible for suppressing NK cell cytotoxicity; the secretomes of different neutrophil subtypes warrant further investigation.

### Both N1-Like and N2-Like Neutrophils Inhibit the Infiltration of NK-92MI Cells into PANC-1 Tumor Spheroids

Besides tumor cytotoxicity, we also measured tumor infiltration as an end-point behavior of NK cells using 3D confocal imaging of the NNTI-chip fixed at 24 h to further investigate the mechanism by which N1-like neutrophils restored the tumor cytotoxicity of NK-92MI cells (Figure 4D). Since NK cells need to form physical contact (i.e., the immunological synapse) with cancer cells before killing them,^72^ the ability to infiltrate into the tumor can be seen as an indirect indicator of the tumor-killing capacity of NK cells. Moreover, higher NK cell infiltration in tumors is correlated with better clinical outcomes in human cancer patients.^27,84^ Here, we sought to examine whether N1-like neutrophils could enhance NK cell infiltration in tumor spheroids compared to N0 and N2-like neutrophils as a way of restoring NK cell tumor cytotoxicity. NK cell infiltration in the tumor spheroid was quantified as the volume of NK-92MI cells inside the spheroid divided by the total volume of the spheroid, i.e., the percentage of infiltrated NK-92MI cells per tumor spheroid.

We found that NK cell infiltration in tumor spheroids was higher without neutrophils than in the presence of N0 neutrophils (8.04% vs 5.02%, *p* < 0.0001), N1-like neutrophils (8.04% vs 5.87%, *p* < 0.01), or N2-like neutrophils (8.04% vs 5.49%, *p* < 0.01) (Figure 6A,B). Interestingly, there was also no significant difference in NK cell infiltration in tumor spheroids between N0, N1-like, and N2-like neutrophils (5.02% vs 5.87% vs 5.49%, *p* > 0.999) (Figure 6A,B). These results show that all three neutrophil subtypes inhibited NK cell infiltration in tumor spheroids and that N1-like neutrophils did not restore NK cell infiltration in tumor spheroids compared to N0 and N2-like neutrophils. Our earlier well plate-based assay showed that conditioned medium from N1-like neutrophils led to an upregulation of the degranulation marker CD107a and the activation marker IFN-γ by NK-92MI cells than those from N0 and N2-like neutrophils (Figure S1D). Hence, it is possible that antitumor (N1) neutrophils restored the tumor cytotoxicity of NK cells by upregulating their secretion of cytotoxic molecules, rather than by promoting the abundance of infiltrated NK cells in the tumor. The effect of different neutrophil subtypes on the secretion profile of NK cells in response to tumor spheroids was not examined in the NNTI-chip and merits further investigation.

**Figure 6:**
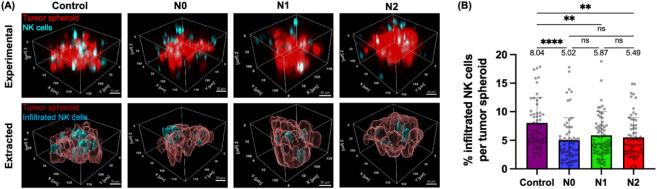
Both N1-like and N2-like neutrophils inhibit the infiltration of NK-92MI cells into PANC-1 tumor spheroids. (A) 3D rendering of representative 10X confocal images showing the infiltration of NK-92MI cells (cyan) in tumor spheroids (red) in the absence (control) or presence of N0, N1-like, or N2-like neutrophils (unstained) in the NNTI-chip fixed at 24 h and the extraction of the volumes of tumor spheroids and infiltrated NK cells by Imaris. Images were acquired as z-stacks with a 2 μm step size. NK cell infiltration was quantified as the volume of infiltrated NK-92MI cells divided by the total spheroid volume. Scale bar, 30 μm. (B) Bar plot showing the percentage of infiltrated NK-92MI cells per tumor spheroid in specified conditions. Each data point represents a spheroid and *n* = 58–77 spheroids per condition. Bars show mean ± SD with mean values written above the points. At least three independent experiments were performed. ns: *p* ≥ 0.05, **p < 0.01, ****p < 0.0001, Kruskal–Wallis test.

## Discussion

In this work, we developed a microphysiological system, NNTI-chip, to quantify the differential effect of N1-like and N2-like human neutrophils on the behaviors of NK cells, specifically migration, motility, tumor cytotoxicity, and tumor infiltration. The NNTI-chip offers a few advantages over traditional *in vitro* and *in vivo* platforms for studying interactions among NK cells, neutrophils, and cancer cells. First, the micropost structures in the NNTI-chip allow flexible spatial organization of different cell types, enabling them to be placed either in three distinct channels (scenario 1) or in the same channel (scenario 2). In scenario 1, the three-channel design enables us to study the migratory preference of NK cells in response to two competing signals from different neutrophil subtypes in the same device. This competitive setup cannot be achieved using transwell chambers, which are the standard tool for studying cell migration.^34,35,37^ Second, live imaging of the NNTI-chip enables real-time visualization and quantitative temporal information on human NK cell migration and tumor spheroid apoptosis with single-cell or single-spheroid resolution, which would be difficult to attain using *in vivo* models and standard end-point molecular biology assays.^36,63,85^ Third, in contrast to the large volumes used in well plates, the reduced volume of microfluidic channels in the NNTI-chip would result in more rapid diffusion and higher concentrations of cell-secreted factors, thereby enhancing the cellular interactions and NK cell responses to be quantified^48,86–89^ (Figure S8). Last but not least, using the NNTI-chip can make imaging of tumor spheroids faster and easier than using well plates. The thick hydrogel in well plates results in variable distances between different tumor spheroids and the objective lens of the microscope, while the small height of microfluidic channels in the NNTI-chip relative to the spheroid diameter helps maintain a nearly uniform focal distance across all tumor spheroids^48^ (Figure S8).

In addition to the development of the microphysiological model, this work also has critical biomedical implications. This study reveals the preference of NK cells to migrate toward N1-like neutrophils over N0 and N2-like neutrophils, suggesting that the antitumor (N1) neutrophil subtype may be more capable of attracting NK cells than the pro-tumor (N2) neutrophil subtype. This finding points to the polarization of tumor-associated neutrophils into an “N1” phenotype to recruit more NK cells into the tumor tissue as a potential therapeutic strategy. We also detected a higher level of NK cell chemokine IP-10 (CXCL10) in the conditioned medium from N1-like neutrophils than in those from N2-like and N0 neutrophils, suggesting that the preferential migration of NK cells toward N1-like neutrophils could be due to their higher level of IP-10 secretion. A previous study by Benigni et al. reports the role of the CXCR3/IP-10 axis in regulating neutrophil-NK cell crosstalk in osteoarthritis.^90^ To confirm the role of the CXCR3/IP-10 axis in the attraction of NK cells toward antitumor (N1) neutrophils, inhibition of this signaling pathway can be performed through genetic knockout of IP-10 in neutrophils or blockade of the IP-10 receptor CXCR3 on NK cells in a future study. This study also shows that naïve (N0) human neutrophils inhibited the tumor cytotoxicity of NK cells, which corroborates previous *in vitro* studies using human cells and mouse cells.^13,27,28^ Importantly, for the first time, this study reveals that antitumor (N1) human neutrophils can restore the tumor-killing capacity of NK cells that was impaired by naïve (N0) and pro-tumor (N2) neutrophils. Taken together, these results show the clinical promise of neutrophil reprogramming to improve not only NK cell recruitment to the tumor tissue but also NK cell cytotoxicity against tumors after recruitment. Nonetheless, N1-like neutrophils in this study only helped NK cells maintain their original level of cytotoxicity against tumors; they did not further enhance it to surpass the original level. Similarly, we showed in our previous work that N1-like neutrophils attenuated tumor progression compared to N2-like neutrophils in terms of invasion, proliferation, and epithelial-mesenchymal transition, but did not reduce the level of tumor progression back to the control condition without neutrophils.^48^ Altogether, these results point to the need for alternative reprogramming approaches to enhance the antitumor power of neutrophils. Recent studies using mouse models have attempted to modify neutrophils to fight cancer in different ways,^91–94^ such as by developing an antibody cocktail to activate neutrophils^91,95^ and by genetically engineering chimeric antigen receptor (CAR)-neutrophils.^92^ In the future, we can use the NNTI-chip to test any synergistic effect of these novel reprogrammed human neutrophils and NK cells on tumor cytotoxicity.

One limitation of this work is the reductionist nature of the engineered biology in the NNTI-chip; the layout of the microfluidic device provides opportunities for recapitulating more physiologically complex scenarios in the future. For example, we can build a vascular structure in the central channel^96–201^ and model the extravasation of NK cells into the tumor tissue in the presence of different neutrophil subtypes. Moreover, the simplified N1/N2 classification fails to encompass the diversity and heterogeneity of neutrophil states in cancer.^19,104^ Recent single-cell RNA sequencing analyses in both mice and human patients have identified various distinct transcriptional states and clusters of neutrophils within tumors that extend beyond the traditional N1/N2 dichotomy.^93,94,105–109^ Thus, future research should consider adopting a more nuanced and refined categorization of neutrophil subpopulations. While the effects of different neutrophil subtypes on NK cell behaviors were shown to be statistically significant in this human cell-based *in vitro* study, whether the effects are biologically significant in humans for therapeutic purposes needs further investigation through *in vivo* studies or clinical trials. Furthermore, the neutrophil-to-NK cell ratio used to assess cytotoxicity against pancreatic tumor spheroids was 2:1, which may be lower than the physiological ratio in human pancreatic tumor tissue. Lastly, the three cell types used in this work (i.e., neutrophils, NK cells, and cancer cells) were human cell lines derived from three different donors, respectively, which could introduce artifacts due to allogeneic reaction.^81^ Using immortalized cell lines also limits the clinical relevance of the study. The NNTI-chip can enable future research using sparse clinical samples of only a few microliters and a few thousand cells,^75^ allowing for the measurement of interactions between patient-derived tumor cells and autologous neutrophils in the presence of drug candidates. This approach can empower personalized medicine and facilitate the translation of novel immunotherapies into cancer patients.^110–112^ The US Food and Drug Administration (FDA) approved the world’s first Investigational New Drug (IND) application for a cancer immunotherapy treatment based solely on organ-on-a-chip data in October 2025. Meanwhile, the US Senate passed the FDA Modernization Act 3.0, which acknowledges microphysiological systems as an alternative to animal models for drug testing in December 2025. Hence, human-relevant complex *in vitro* models such as the NNTI-chip are now ready to serve as useful tools for both mechanistic studies and preclinical testing of novel cancer immunotherapies.^202–205^

## Conclusions

In summary, we engineered a microphysiological system using human cells to quantitatively investigate the distinct effects of antitumor N1-like and pro-tumor N2-like neutrophil subtypes on NK cell behaviors including migration, motility, tumor cytotoxicity, and tumor infiltration using both real-time and end-point measurements. We found that NK cells showed preferential migration toward N1-like neutrophils over N2-like neutrophils, although they showed lower motility in speed, displacement, and directionality after migration toward N1-like neutrophils in comparison to N2-like neutrophils. Moreover, N1-like neutrophils reversed the immunosuppression mediated by N2-like neutrophils by restoring the cytotoxicity against pancreatic tumor spheroids of NK cells to their original level. Interestingly, both N1-like and N2-like neutrophils inhibited NK cell infiltration into tumor spheroids. This study reveals the multifaceted role of human neutrophils in modulating NK cell behaviors and the complex crosstalk between different immune cell types, suggesting the reprogramming of tumor-associated neutrophils to enhance NK cell recruitment and cytotoxicity as a potential immunotherapy strategy for pancreatic cancer. In the future, the microphysiological system can be used to study the effect of neutrophils on NK cell extravasation into the tumor tissue and test novel drug candidates by using patient-derived tumor organoids and autologous immune cells for precision medicine.

## Methods

### Cell Culture

The human natural killer cell line NK-92MI (American Type Culture Collection ATCC, CRL-2408) was cultured in Roswell Park Memorial Institute (RPMI) 1640 (Corning, 10040CV) supplemented with 10% fetal bovine serum (FBS, Corning, 35015CV), 1% sodium pyruvate (Gibco, 11360070), 1% minimum essential medium (MEM) nonessential amino acids solution (Gibco, 11140050), 1% GlutaMAX (Gibco, 35050061), 0.1% 2-mercaptoethanol (Gibco, 21985023), and 1% penicillin–streptomycin (Cytiva), denoted as complete RPMI. The red fluorescent protein (RFP)-expressing human pancreatic cancer cell line RFP-PANC-1 (FenicsBIO, CL-1191) was cultured in Dulbecco’s Modified Eagle’s Medium (DMEM, ATCC) containing 10% fetal bovine serum (FBS, ATCC) and 1% penicillin–streptomycin (ATCC), designated as complete DMEM. For subculturing, RFP-PANC-1 cells (hereafter referred to as PANC-1 cells) were dissociated using TrypLE Express Enzyme (Gibco) by incubation for 2 min at 37 °C. The human promyelocytic leukemia cell line HL-60 (ATCC, CCL-240) was maintained in Iscove’s Modified Dulbecco’s Medium (IMDM, ATCC) containing 10% FBS and 1% penicillin–streptomycin, designated as complete IMDM. All cell lines were routinely incubated at 37 °C under 5% CO_2_ conditions.

### Generation of Human Neutrophil Subtypes

HL-60 cells between passages 7 and 14 were seeded at a concentration of 2 × 10^5^ cells/mL in T-25 culture flasks (10 mL/flask). To induce neutrophil-like differentiation, cells were treated with 1.5% dimethyl sulfoxide (DMSO, ATCC) for 4 days, generating neutrophil-like HL-60 cells (dHL-60 neutrophils).^63–65^ Following differentiation, dHL-60 neutrophils were polarized for 24 h into different subtypes. As previously described, an N1-like (antitumor) phenotype was induced by treating with 100 ng/mL lipopolysaccharide (LPS, Sigma-Aldrich, L2630), 50 ng/mL interferon-β (IFN-β, R&D Systems, 8499-IF-010/CF), and 50 ng/mL interferon-γ (IFN-γ, R&D Systems, 285-IF-100/CF).^18^ An N2-like (pro-tumor) phenotype was induced with 100 ng/mL transforming growth factor-β (TGF-β, Sigma-Aldrich, T7039).^66,67^ For the unpolarized control group (N0), dHL-60 neutrophils were incubated for 24 h with an equivalent volume of phosphate-buffered saline (PBS, Gibco) as the vehicle control. Both differentiation and polarization procedures were conducted at 37 °C in a humidified incubator with 5% CO_2_. Surface marker expression of different neutrophil subtypes was validated as shown in Figure S1B.

### Generation of 3D Tumor Spheroids

PANC-1 cells between passages 5 and 20 were used for the formation of tumor spheroids. Tumor spheroids were established using the Elplasia round-bottom ultralow attachment (ULA) 6-well plate (Corning, 4440) following the manufacturer’s instructions. Prior to cell seeding, each well was added with 1.5 mL of complete DMEM containing 1% Matrigel (Corning, 354277) and centrifuged to eliminate air trapped within the microcavities. Subsequently, ∼86,550 PANC-1 cells suspended in 1.5 mL of complete DMEM containing 1% Matrigel were added to each well, corresponding to ∼30 cells per microcavity (2,885 microcavities per well), to promote spheroid assembly.^51,52^ The plate was incubated for 48 h at 37 °C under a humidified atmosphere with 5% CO_2_, allowing the formation of a single spheroid per microcavity. The resulting spheroids exhibited an average diameter of 93.0 ± 18.8 μm (Figure 4C).

### Design and Fabrication of the NNTI-Chip

The NNTI-chip comprises two side channels A and B for loading dHL-60 neutrophils (N0, N1-like, or N2-like) and PANC-1 tumor spheroids (9 mm long, 700 μm wide) embedded in collagen hydrogel, which mimics the extracellular matrix of the tumor tissue, a central channel for loading NK-92MI cells embedded in collagen hydrogel (9 mm long, 400 μm wide), and two medium channels, each having two medium reservoirs for loading culture medium (6.7 mm long, 1 mm wide) (Figure 1A). The five channels in parallel are ∼148 μm in height as measured by a profilometer; they are separated by four rows of 16 trapezoidal posts (base lengths of 200 and 84 μm, height of 100 μm, interpost spacing of 50 μm).^48^ This geometric configuration creates a balance between surface tension and capillary forces that facilitates hydrogel confinement within the designated channels.^75^ The NNTI-chip was made using standard microfabrication techniques as previously described.^48^ Briefly, a silicon wafer master mold was first fabricated by using photolithography in the cleanroom. Then, the microfluidic chips were fabricated using polydimethylsiloxane (PDMS, Sylgard 184, Dow Corning) with standard soft lithography. After air plasma treatment, six devices were attached to a glass-bottom 6-well plate (Cellvis, P061.5HN) and incubated at 80 °C overnight to promote bonding and restore hydrophobicity.^113^ The chips were sterilized under UV light for 30 min before cell seeding.

### NK Cell Migration toward Neutrophil Subtypes in the NNTI-Chip

To maintain a humidified environment and reduce evaporation, 1 mL of cold sterile water was added around each chip on the glass-bottom 6-well plate. For the preparation of a 1.2 mg/mL collagen gel solution (200 μL total volume, pH 7.4), 80 μL of 3 mg/mL rat-tail type I collagen (Corning, 354236) was mixed on ice with 20 μL of 10× PBS, 96 μL of sterile deionized water, and 4 μL of 0.5 N NaOH (Fisher Chemical). The pH of the final mixture was verified to be approximately 7.4 using pH indicator paper. For the competitive scenario (scenario 1), ∼30,000 N0, N1-like, or N2-like dHL-60 neutrophils (5 × 10^6^ cells/mL) in 6 μL of collagen gel solution was loaded into side channels A and B of the NNTI-chip on ice without staining. For the noncompetitive scenario (scenario 0), 6 μL of collagen gel solution without cells was introduced into side channel B on ice, serving as a negative control. The NNTI-chips were subsequently incubated at 37 °C with 5% CO_2_ for 30 min to allow gel polymerization. Following gelation, each of the two medium channels was supplemented with 100 μL of complete RPMI to maintain cell viability and prevent dehydration. The devices were then maintained for 24 h at 37 °C with 5% CO_2_ to allow neutrophil secretion of NK cell-attracting chemokines prior to the introduction of NK-92MI cells into the central channel. During the 24 h of incubation, the central channel remained unfilled (air-filled) to establish the gradients of neutrophil-secreted chemokines needed for NK cell migration. NK-92MI cells from passages 5 to 22 were stained with Vybrant DiD dye (1:1000, Invitrogen, V22887) in serum-free RPMI at 37 °C for 10 min and washed once with complete RPMI to stop the staining.^62^ Following the 24 h incubation period, ∼35,000 NK-92MI cells (5 × 10^6^ cells/mL) suspended in 7 μL of collagen gel solution were introduced on ice into the central channel. Following the 30 min gel polymerization step, time-lapse imaging of the chips was initiated immediately to monitor the migration and motility of NK-92MI cells into the side channels. To acquire representative images (Figure 1B), NK-92MI cells were stained with Vybrant DiO dye (1:500, Invitrogen, V22886) in serum-free RPMI at 37 °C for 10 min, N1-like neutrophils with CellTracker Orange CMRA dye (10 μM, Invitrogen, C34551) in serum-free IMDM for 20 min, and N2-like neutrophils with Vybrant DiD dye (1:1000) in serum-free IMDM for 20 min.

### NK Cell Cytotoxicity against Tumor Spheroids in the Presence of Neutrophil Subtypes in the NNTI-Chip

NK-92MI cells from passages 5–25 were stained with Vybrant DiD dye in serum-free RPMI at 37 °C for 10 min and washed once with complete RPMI to stop the staining.^62^ PANC-1 tumor spheroids were harvested from the ULA plate after 48 h of culture. To minimize spheroid loss during handling, all pipet tips used to transfer tumor spheroids were precoated with 2% bovine serum albumin (BSA, Sigma-Aldrich). N0, N1-like, and N2-like dHL-60 neutrophils after 24 h of polarization were harvested for subsequent experiments. To acquire representative images (Figure 4B), neutrophils were stained with 5 μM CellTracker Violet BMQC dye (Invitrogen, C10094) in serum-free IMDM at 37 °C for 30 min,^76,114^ and washed once with complete IMDM to terminate staining. For the control conditions without NK-92MI cells, neutrophils were stained with Vybrant DiD (1:1000) in serum-free IMDM for 20 min. Neutrophils were left unstained in the main experiments involving NK-92MI cells. One mL of cold sterile water was dispensed around each NNTI-chip on the glass-bottom 6-well plate to maintain humidity and minimize evaporation. A collagen gel solution was prepared using the method detailed in the previous section. A mixture of ∼11 tumor spheroids, ∼18,000 NK-92MI cells (3 × 10^6^ cells/mL), and ∼36,000 dHL-60 neutrophils (6 × 10^6^ cells/mL) in 6 μL of collagen gel solution was seeded into each of the two side channels of the NNTI-chip on ice to mimic the tumor tissue. Tumor spheroids alone, tumor spheroids with NK-92MI cells without neutrophils, and tumor spheroids with neutrophils without NK-92MI cells in gel were seeded into the NNTI-chips as different controls for assessing tumor spheroid apoptosis. The NNTI-chips were incubated for 30 min at 37 °C with 5% CO_2_ to allow gel polymerization. Subsequently, 100 and 7 μL of complete RPMI were added to the two medium channels and the central channel, respectively, to maintain cell viability and hydration. Complete RPMI was supplemented with 2 μM CellEvent Caspase-3/7 Green (Invitrogen, C10423) prior to loading into the chips to monitor tumor spheroid apoptosis.^75,81,82^ To reduce water evaporation from the NNTI-chip, 20 μL of mineral oil (Sigma-Aldrich) was used to overlay the culture medium in each medium reservoir.^115^

### Time-Lapse Imaging and Analysis

Time-lapse live imaging of six NNTI-chips was performed concurrently on a fully automated Nikon ECLIPSE Ti2-E microscope using a Plan Apo 10X objective (NA = 0.45) at 37 °C with 5% CO_2_ for 24 h. In scenario 1, images were acquired using NIS-Elements (Nikon Inc.) software and recorded using brightfield and Cy5 channels at 2 h intervals for 24 h to capture NK-92MI cell migration from the central channel into side channels A and B housing neutrophil subtypes. Images were also acquired every 2 min for 30 min at 6 h intervals for 24 h to enable cell tracking and measurement of NK cell motility after migration into the side channels.^96^ For scenario 2, images were taken over 24 h at 2 h intervals to capture the apoptosis of tumor spheroids in real time and recorded using brightfield, mCherry (tumor spheroids), Cy5 (NK-92MI cells or neutrophils), and FITC (apoptotic cells) channels.

To quantify NK cell migration to neutrophils in scenario 1, we measured the percentage of NK-92MI cells that migrated into either side channel A or B. Briefly, cells present in side channel A or B every 2 h were quantified using the TrackMate plugin in ImageJ (National Institutes of Health). The counted cell number was normalized to the initial number of cells in the central channel at *t* = 0 h.^96^ The rate of NK cell migration was determined by calculating the hourly increase in the percentage of migration at each time point. To quantify NK cell motility in the side channels in scenario 1, automatic tracking of cells was conducted on Cy5 fluorescence images over each duration of 30 min using TrackMate.^116,117^ The cell motility parameters extracted for statistical analysis included speed, displacement (Euclidean distance), and directionality (linearity of forward progression; Figure 3B). Only tracks whose mean speed was higher than 0.20 μm/min were used for analysis to exclude nonmotile cells.

To quantify the temporal dynamics of tumor spheroid apoptosis in scenario 2, we measured the normalized fluorescence intensity of Caspase-3/7 Green per tumor spheroid. Briefly, the boundary of each tumor spheroid at each time point was defined by the thresholding of mCherry signals in ImageJ, and the mean FITC intensity within the boundary was measured. The normalized apoptosis of a tumor spheroid at a given time point was calculated as the difference between the intensity at that time point and the initial intensity (*t* = 0 h) divided by the initial intensity. The rate of apoptosis was calculated as the increase in the level of normalized tumor spheroid apoptosis per hour at a given time point.

### Confocal Imaging and Analysis

To measure NK cell infiltration in tumor spheroids and the end-point spheroid apoptosis in 3D, the NNTI-chips were fixed immediately after 24 h of time-lapse imaging using 4% paraformaldehyde (PFA, Electron Microscopy Sciences) for 15 min at room temperature. Devices were subsequently filled with PBS and stored at 4 °C, protected from light until confocal imaging. All reagents were introduced and removed via the medium reservoirs of the devices in 100 μL volumes. Imaging was performed using a spinning disk confocal microscope (Nikon CSU-W1) with a PLAN APO 10X (NA = 0.45) air objective. Z-stack images were acquired at 2 μm step intervals using NIS-elements software (AR 5.42.04), spanning a total depth of ∼80–120 μm. Tumor spheroids were captured on the mCherry channel, DiD-stained NK-92MI cells or neutrophils on the Cy5 channel, and apoptotic cells were captured on the FITC channel.

Tumor spheroid apoptosis was calculated as the ratio of the apoptotic volume to the total spheroid volume.^76,118^ The apoptotic volume was identified from FITC fluorescence signals, whereas the total spheroid volume was identified from mCherry fluorescence signals, both with the surface function in Imaris 10.2.0 (Oxford Instruments). To improve the accuracy of quantification, FITC signals colocalized with Cy5 signals were attributed to apoptotic NK-92MI cells and were excluded from the analysis. To evaluate NK cell infiltration, the volume of all NK-92MI cells located within the reconstructed spheroid surface was determined based on Cy5 signals with the surface function of Imaris and then normalized by the total volume of the spheroid. 3D rendering of both experimental and extracted versions of representative confocal images was created in Imaris. For consistency, identical brightness and contrast parameters were applied across all of the experimental groups within each fluorescence channel.

### Immunofluorescence Staining and Analysis

To validate the identity of NK-92MI cells in the NNTI-chip, the cells prelabeled with Vybrant DiD dye were immunostained for the typical NK cell marker CD56.^49^ In parallel experiments, to validate the polarization states of neutrophil subtypes in the NNTI-chip, N0, N1-like, and N2-like neutrophils prelabeled with Vybrant DiD dye were immunostained for the typical N1 marker CD54 (ICAM-1).^1,18^ Immunofluorescence staining in the NNTI-chip was conducted following previously established protocols.^46,48,113^ Briefly, cells were washed twice with PBS and fixed for 15 min at room temperature with 4% PFA at *t* = 0 and *t* = 24 h of on-chip culture. After two additional PBS washes, cells were blocked with 10% goat serum (Invitrogen, 50062Z) for 2 h at room temperature to minimize nonspecific binding. NK-92MI cells were incubated at 4 °C overnight with the fluorescently conjugated antibody FITC antihuman CD56 (1:50, BioLegend, 304603) diluted in 10% goat serum. Neutrophils were similarly incubated at 4 °C overnight with the following fluorescently conjugated antibody diluted in 10% goat serum: Brilliant Violet 421 antihuman CD54 (1:100, BioLegend, 353131). After two PBS washes, the NNTI-chips were incubated with PBS at 4 °C for 24 h on a rocker to ensure thorough removal of unbound antibody. All reagents were introduced and removed via the medium reservoirs of the chips in 100 μL volumes. The NNTI-chips were imaged with a Nikon ECLIPSE Ti2-E epifluorescence microscope using a Plan Apo 20X objective (NA = 0.80). NK-92MI cells were acquired on the Cy5 channel and CD56 on the FITC channel. Neutrophils were acquired on the Cy5 channel and CD54 on the DAPI channel. To measure marker expression per cell, we defined the boundary of each cell by thresholding of Cy5 signals and measured the mean fluorescence intensity within the boundary on the FITC or DAPI channel in ImageJ. The same brightness and contrast settings were applied to images of all experimental conditions for each channel when representative images were created in ImageJ.

### Flow Cytometry

To validate the success of neutrophil polarization, flow cytometry was performed to assess surface marker expression across different neutrophil subtypes. Following polarization, N0, N1-like, and N2-like dHL-60 neutrophils were harvested and analyzed for typical N1 markers CD11b^high^, CD54 (ICAM-1)^high^, and CD62L (L-selectin)^low^, and for the typical N2 marker CD182 (CXCR2)^high^.^18^ Cells were first washed once with PBS and stained using the Zombie R685 Fixable Viability Kit (BioLegend, 423119) for 10 min at room temperature to label dead cells. After a wash with the flow cytometry staining buffer (Invitrogen, 00422226), samples were stained for 20 min at 4 °C with the following fluorescently conjugated antibodies diluted in the staining buffer: Alexa Fluor 488 antihuman CD11b (1:50, BioLegend, 301317), Brilliant Violet 421 antihuman CD54 (1:50, BioLegend, 353131), Brilliant Violet 605 antihuman CD62L (1:50, BioLegend, 304833), and PE antihuman CD182 (1:50, BioLegend, 320706). Following an additional wash with the staining buffer, cells were fixed for 10 min with 4% PFA, resuspended in 200 μL of staining buffer, and stored at 4 °C protected from light until acquisition on an LSR Fortessa flow cytometer (BD Biosciences).

To examine the effect of different neutrophil subtypes on NK cell cytotoxicity, we performed flow cytometry to measure the expression levels of the degranulation marker CD107a and the activation marker IFN-γ by NK-92MI cells.^72–74^ After polarization, N0, N1-like, and N2-like dHL-60 neutrophils were seeded at a density of 10^6^ cells/mL in 1 mL of complete RPMI per well on a 24-well plate and cultured for 24 h. The supernatants or conditioned media from N0, N1-like, and N2-like neutrophils were then collected and centrifuged at 2000 × g for 10 min at 4 °C to remove cellular debris. The conditioned media were frozen at −80 °C for up to 1 month before use. NK-92MI cells at a density of 10^6^ cells/mL were treated with 100 μL of N0, N1, or N2 neutrophil-conditioned medium per well on a V-bottom 96-well plate for 6 h. NK-92MI cells were then stimulated with 20 nM phorbol 12-myristate 13-acetate (PMA, Sigma-Aldrich, P8139) and 0.1 μg/mL ionomycin (Sigma-Aldrich, I0634) for 4 h in the incubator in the presence of protein transport inhibitors GolgiPlug and GolgiStop (1:1000, BD Biosciences) for intracellular accumulation of IFN-γ. The stimulation was performed also in the presence of FITC antihuman CD107a (1:100, Biolegend, 328606).^119^ After one PBS wash, NK-92MI cells were stained with a Zombie R685 Fixable Viability Kit for 10 min at room temperature to label dead cells. Following one PBS wash, cells were fixed and permeabilized with the eBioscience Foxp3/Transcription Factor Staining Buffer Set (Invitrogen, 00–5523–00) for 30 min at room temperature. After washing with the permeabilization buffer once, cells were stained with PE/Cyanine7 antihuman IFN-γ (1:100, Biolegend, 502527) for 30 min at room temperature. Cells were washed with the permeabilization buffer once and resuspended in the flow cytometry staining buffer and analyzed by a BD FACSymphony A3 flow cytometer.

Flow cytometry data were processed using FlowJo 10.8.1 (BD Biosciences). The gating strategy was applied sequentially. First, cells were selected based on the side scatter area (SSC-A, cell granularity) versus forward scatter area (FSC-A, cell size) density plot to eliminate the debris. Next, single cells were selected by gating on the forward scatter height (FSC-H) versus forward scatter area (FSC-A) density plot to remove cell doublets and aggregates. Finally, live cells were selected as Zombie-dye-negative events, thereby excluding dead cells from further analysis.

### Enzyme-Linked Immunosorbent Assay (ELISA) and Luminex

The secretion levels of IP-10 (CXCL10), IL-12, IL-15, IL-18, TNF-α, and IFN-γ by different neutrophil subtypes were examined by using ELISA or Luminex. After polarization, N0, N1-like, and N2-like dHL-60 neutrophils were seeded at a density of 10^6^ cells/mL in 1 mL of complete RPMI per well on a 24-well plate and cultured for 24 h. The supernatants or conditioned media from N0, N1-like, and N2-like neutrophils were then collected and centrifuged at 2000 × g for 10 min at 4 °C to remove cellular debris. The conditioned media were frozen at −80 °C for up to 1 month until analyzed. Concentrations of IP-10 in the conditioned media were measured using the human IP-10 ELISA kit (BioLegend, 439907) according to the manufacturer’s protocol. The conditioned medium from N1-like neutrophils was diluted 10 times before testing, while those from N0 and N2-like neutrophils were tested without dilution. Absorbance at 450 nm was measured by using a microplate reader (BioTek Epoch 2). Standard curves were generated, and sample concentrations were interpolated using Prism 9 software (GraphPad) based on the Richard’s five-parameter logistic regression model. Concentrations of NK cell-activating cytokines IL-12, IL-15, IL-18, TNF-α, and IFN-γ ^23,70,71^ in the conditioned media were measured using the Human Luminex Discovery Assay kit (R&D Systems, LXSAHM-05) according to the manufacturer’s protocol.

### Statistical Analysis

For each assay, at least three independent experiments were conducted unless otherwise stated. Statistical analyses were carried out using Prism 9 (GraphPad). Data were presented as mean ± standard deviation (SD) in the graphs. Details of the specific statistical tests applied are provided in the corresponding figure legends. Normality was assessed using the Shapiro–Wilk test. For data sets that did not meet normal distribution assumptions, nonparametric analyses (Kruskal–Wallis test or Mann–Whitney test) were performed. For normally distributed data, parametric tests (one-way ANOVA or unpaired Student’s *t* test) were performed. Statistical significance was defined as *p* < 0.05.

## Supplementary Material







## Notes

Figure S1: Generation and characterization of N0, N1-like, and N2-like dHL-60 neutrophils. Figure S2: NK-92MI cells maintain their NK cell identity in the NNTI-chip. Figure S3: N1-like and N2-like neutrophils maintain their polarization states in the NNTI-chip in scenario 1. Figure S4: NK-92MI cells show greater migration toward N1-like neutrophils than N2-like neutrophils in a noncompetitive scenario. Figure S5: NK-92MI cells show lower motility after migration toward N1-like neutrophils than N2-like neutrophils at *t* = 6, 18, and 24 h. Figure S6: N1-like and N2-like neutrophils maintain their polarization states in the NNTI-chip in scenario 2. Figure S7: N1-like and N2-like neutrophils do not affect the apoptosis of PANC-1 tumor spheroids in the absence of NK-92MI cells. Figure S8: Advantages of the NNTI-chip over traditional well plate-based cell culture systems for immune cell-tumor coculture. Table S1: The change and percentage change of the effect of N1-like and N2-like neutrophil subtypes on key NK cell behaviors measured in the NNTI-chip (PDF)
Video S1: The motility of NK-92MI cells postmigration in the NNTI-chip at *t* = 6, 12, 18, and 24 h of time-lapse imaging and the extraction of single-cell trajectories (AVI)
Video S2: The temporal dynamics of tumor spheroids undergoing apoptosis in the presence of NK-92MI cells over 24 h of time-lapse imaging (AVI)
