# Supplementary material for: Quantifying the Dual Effect of Antitumor and Pro-Tumor Human Neutrophils on Natural Killer Cell Behaviors in a Microphysiological System
Source: ACS Biomater Sci Eng. 2026 Mar 18;12(4):2479–95. doi: 10.1021/acsbiomaterials.5c02083 (PMC13080770; doi:10.1021/acsbiomaterials.5c02083)
Supplement: Supplementary file 1 [file ab5c02083_si_001.pdf]

**Supporting Information**

# Quantifying the dual effect of anti-tumor and pro-tumor human neutrophils on natural killer cell behaviors in a microphysiological system

**Shuai Shao[1,2], Caroline N. Jones[1,2 *]**

[1] Department of Bioengineering, The University of Texas at Dallas, Richardson, TX, 75080, USA

[2] Department of Biomedical Engineering, UT Southwestern Medical Center, Dallas, TX, 75235, USA

[*] email: caroline.jones@utdallas.edu

## Supplementary Figures

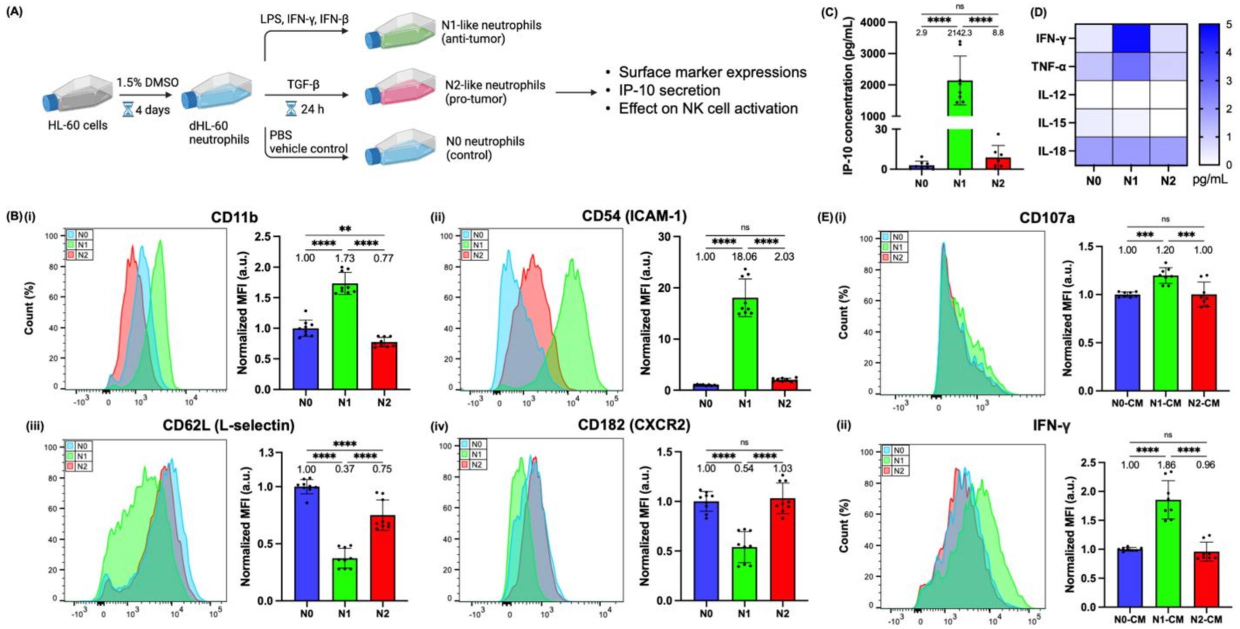


**Figure S1. Generation and characterization of N0, N1-like, and N2-like dHL-60 neutrophils. (A)** HL-60 cells were differentiated with 1.5% DMSO for 4 days into a neutrophil-like state and polarized for 24 h with 100 ng/mL LPS, 50 ng/mL IFN-γ, and 50 ng/mL IFN-β towards an N1-like state or with 100 ng/mL TGF-β toward an N2-like state. The unpolarized control was denoted as N0 neutrophils. Created with BioRender.com. **(B)** Expressions of surface markers CD11b **(i)**, CD54 (ICAM-1) **(ii)**, CD62L (L-selectin) **(iii)**, and CD182 (CXCR2) **(iv)** by N0, N1-like, and N2-like neutrophils were measured using flow cytometry. Histograms from one representative experiment (left panel) and the median fluorescence intensity (MFI) normalized by the mean of the N0 condition from three independent experiments (right panel) are shown. Bars show mean ± SD with the mean values written above the points. **(C)** Concentrations of IP-10, an NK cell chemoattractant, in conditioned media (CM) from N0, N1-like, and N2-like neutrophil cultures were measured by ELISA. CM samples were collected from four independent experiments. **(D)** Concentrations of NK cell-activating cytokines TNF-α, IFN-γ, IL-12, IL-15, and IL-18 in conditioned media (CM) from N0, N1-like, and N2-like neutrophil cultures were measured by Luminex. CM samples were collected from one experiment. **(E)** Expressions of degranulation marker CD107a **(i)** and activation marker IFN-γ **(ii)** by NK-92MI cells after treatment with CM from N0, N1-like, and N2-like neutrophil cultures were measured using flow cytometry. Marker expressions were induced by PMA and ionomycin in the presence of protein transport inhibitors GolgiPlug and GolgiStop to keep IFN-γ within NK-92MI cells. Histograms from one representative experiment (left panel) and the median fluorescence intensity (MFI) normalized by the mean of the N0 condition from four independent experiments (right panel) are shown. ns: ≥0.05, *: $p<0.05$, **: $p<0.01$, ****: $p<0.0001$, ANOVA with Tukey multiple comparisons test.

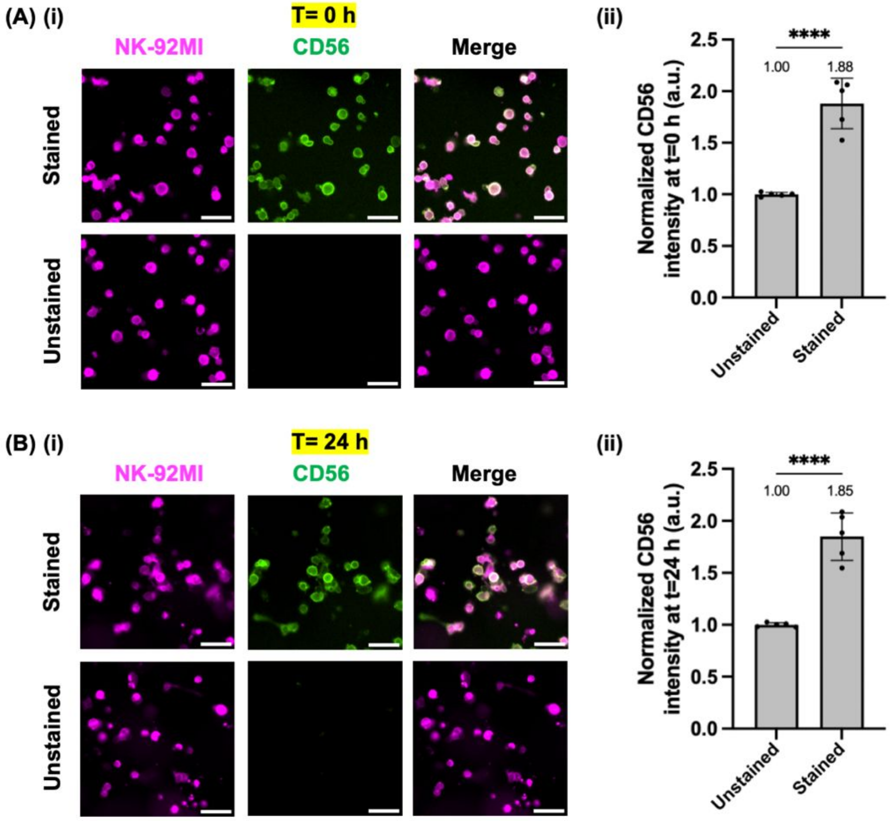


**Figure S2. NK-92MI cells maintain their NK cell identity in the NNTI-chip.** NK-92MI cells express typical NK cell marker CD56 throughout 24 h of on-chip culture. **(A) (i)** Representative 20X epifluorescence images of NK-92MI cells (DiD, magenta) immunostained for CD56 (green) at t=0 h after being embedded in collagen hydrogel and loaded into the NNTI-chip. Scale bar, 50 µm. **(ii)** Bar plot showing the normalized CD56 intensity of NK-92MI cells at t=0 h. Each data point represents the mean value of all cells per ROI. n = 5 ROIs per condition. Raw values were normalized by the mean of the unstained control condition. Bars show mean ± SD with the mean values written above the points. **(B) (i)** Representative 20X epifluorescence images of NK-92MI cells (DiD, magenta) immunostained for CD56 (green) at t=24 h after being embedded in collagen hydrogel and loaded into the NNTI-chip. Scale bar, 50 µm. **(ii)** Bar plot showing the normalized CD56 intensity of NK-92MI cells at t=24 h. One experiment was performed. ****: $p<0.0001$, unpaired t test.

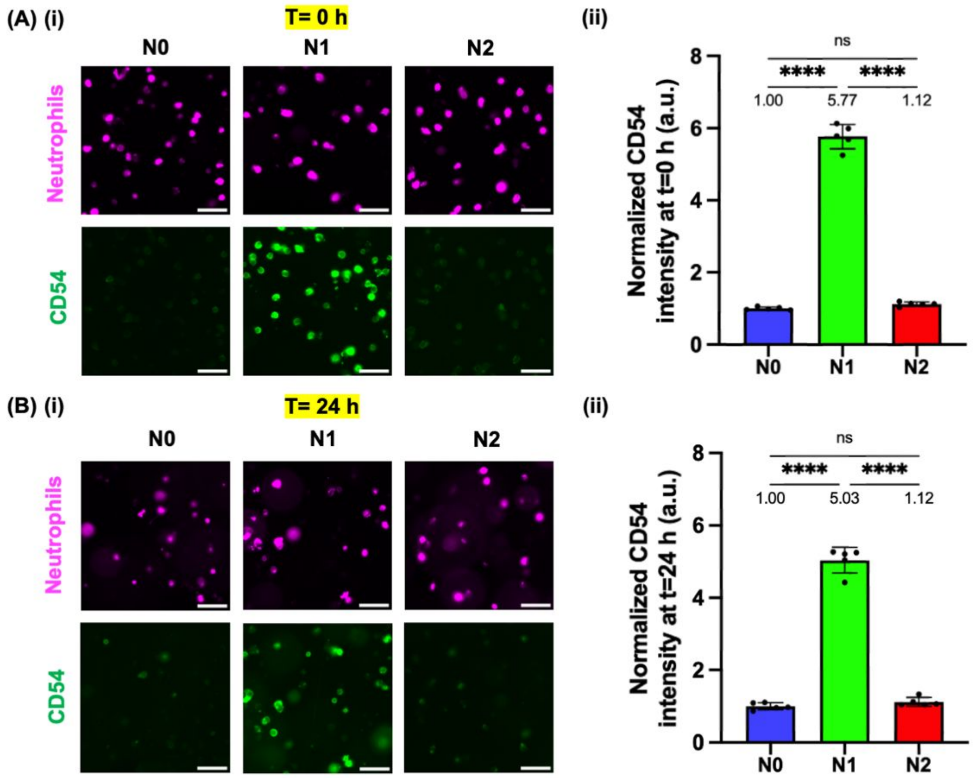


**Figure S3. N1-like and N2-like neutrophils maintain their polarization states in the NNTI-chip in scenario 1.** N1-like neutrophils expressed a significantly higher level of typical N1 marker CD54 than N2-like neutrophils throughout 24 h of on-chip culture. **(A) (i)** Representative 20X epifluorescence images of N0, N1-like, and N2-like neutrophils (DiD, magenta) immunostained for CD54 (ICAM-1) (blue) at t=0 h after being embedded in collagen hydrogel and loaded into the NNTI-chip. Scale bar, 50 µm. **(ii)** Bar plot showing the normalized CD54 intensity of N0, N1-like, and N2-like neutrophils at t=0 h. Each data point represents the mean value of all cells per ROI. Raw values were normalized by the mean of the N0 condition. n = 5 ROIs per condition. Bars show mean ± SD with the mean values written above the points. **(B) (i)** Representative 20X epifluorescence images of N0, N1-like, and N2-like neutrophils (DiD, magenta) immunostained for CD54 (ICAM-1) (blue) at t=24 h after being embedded in collagen hydrogel and loaded into the NNTI-chip. Scale bar, 50 µm. **(ii)** Bar plot showing the normalized CD54 intensity of N0, N1-like, and N2-like neutrophils at t=24 h. One experiment was performed. ns: ≥0.05, ****: p<0.0001, ANOVA with Tukey multiple comparisons test.

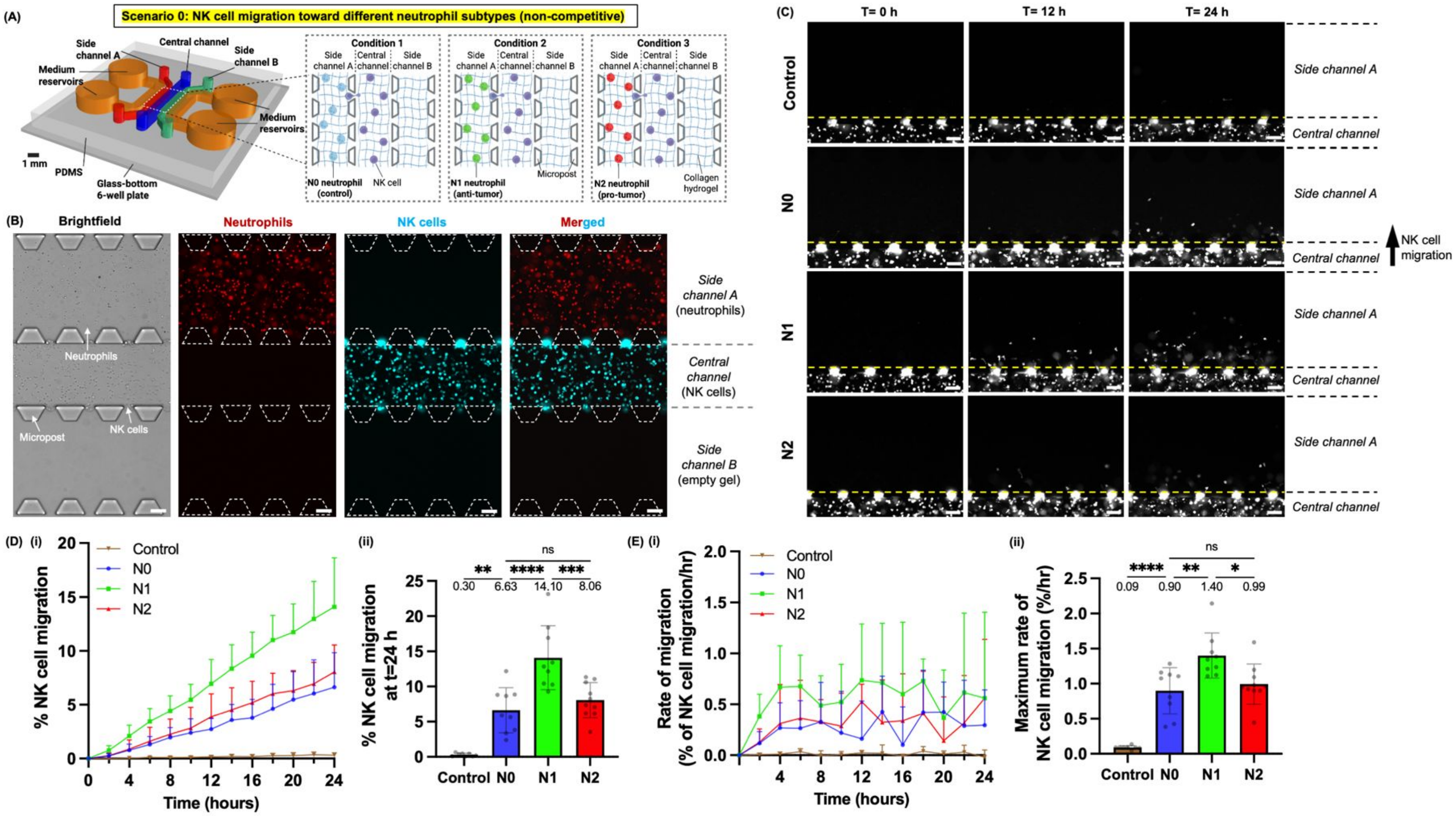


**Figure S4. NK-92MI cells show greater migration toward N1-like neutrophils than N2-like neutrophils in a non-competitive scenario. (A)** The NTI-chip models the migration of NK cells from the central channel into side channel A housing different human neutrophil subtypes (i.e., N0, N1, or N2 neutrophils). Both neutrophils and NK cells were embedded in 3D collagen hydrogel to mimic the extracellular matrix of the tumor tissue. Side channel B housed empty hydrogel, acting as a negative control for NK cell migration. Created with Rhino 7 and BioRender.com. **(B)** Representative 10X brightfield and epifluorescence images showing neutrophils (red) in side channel A and NK cells (blue) in the central channel immediately after loading of NK cells into the NTI-chip. The three channels are separated and interconnected by PDMS microposts. Scale bar, 100 µm. **(C)** Representative 10X images showing NK-92MI cells (white) migrating from the central channel into side channel A housing empty gel control, N0 neutrophils, N1-like neutrophils, or N2-like neutrophils (unstained) at t=0 h, 12 h, and 24 h as representative time points. The yellow dashed line marks the boundary between the central channel and side channel A. Scale bar, 100 µm. **(D) (i)** Line graphs showing the percentage of NK cell migration, defined as the number of NK-92MI cells in side channel A at a given time point divided by the initial number of NK-92MI cells in the central channel at t=0 h, every 2 h over 24 h in specified conditions. Bars show mean + SD. **(ii)** Bar plot showing the percentage of NK cell migration at t=24 h. Bars show mean ± SD with mean values written above the points. Each data point represents an NTI-chip and n= 9-10 chips per condition. **(E) (i)** Line graphs showing the rate of NK cell migration, defined as the increase in the percentage of NK cell migration per hour, every 2 h over 24 h in specified conditions. Bars show mean + SD. **(ii)** The maximum rate of NK cell migration, defined as the highest rate of NK cell migration at any time point over 24 h, in specified conditions. Five independent experiments were performed. ns: $p>0.05$, *: $p<0.05$, **: $p<0.01$, ***: $p<0.001$, ****: $p<0.0001$, ANOVA with Tukey multiple comparisons test.

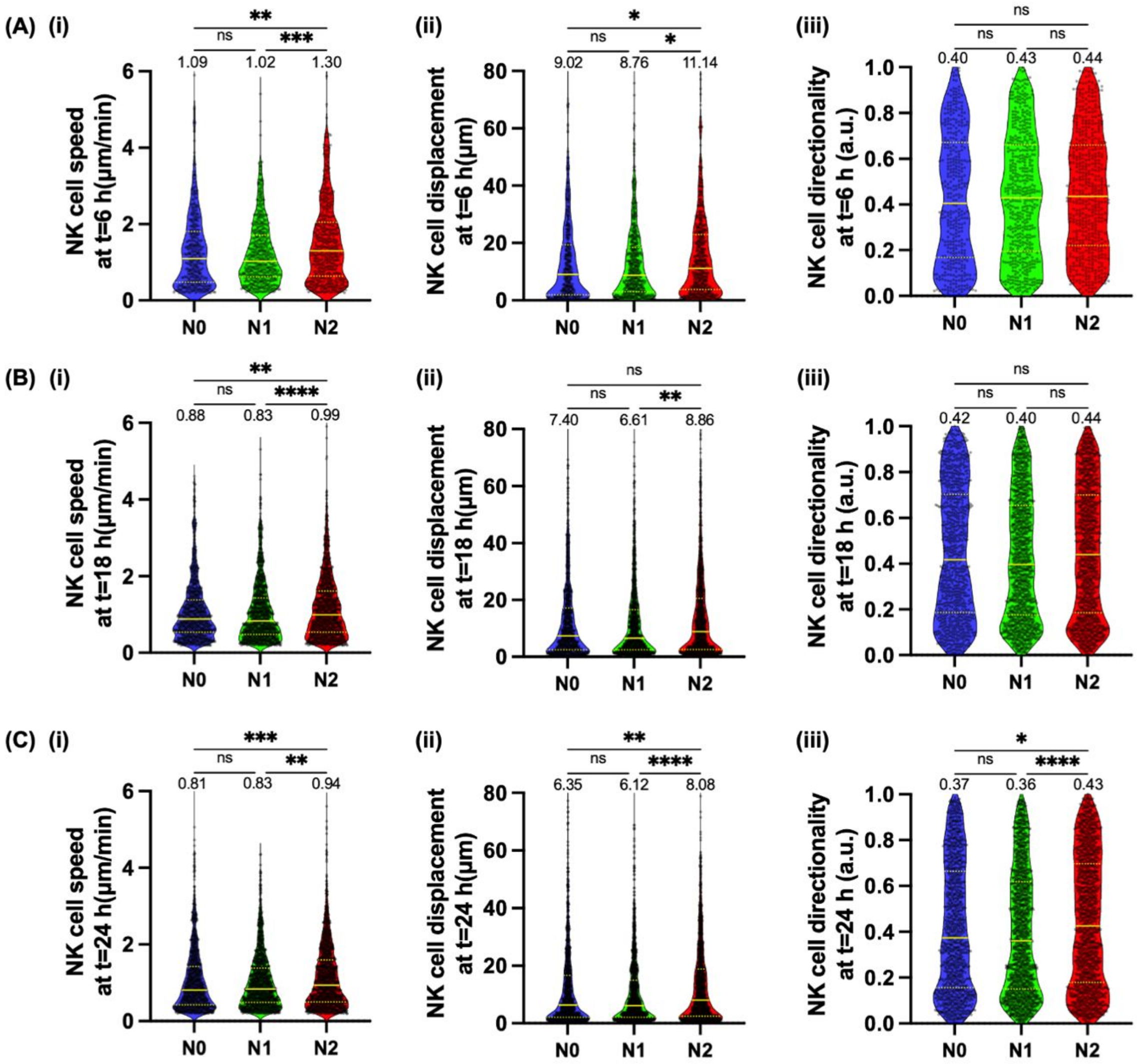


**Figure S5. NK-92MI cells show lower motility after migration toward N1-like neutrophils than N2-like neutrophils at t=6 h, 18 h, and 24 h.** Violin plots showing the speed **(i)**, displacement **(ii)**, and directionality **(iii)** of motile NK-92MI cells with single-cell resolution at t= 6 h **(A)**, 18 h **(B)**, and 24 h **(C)** after after migration toward N0, N1-like, and N2-like neutrophils. Each data point represents a single NK-92MI cell and n= 383-1580 cells from 17-18 side channels of the NTI-chips per condition. The yellow solid lines show the medians and the yellow dashed lines show 25% and 75% percentiles. Median values are written above the points. Five independent experiments were performed. ns: ≥0.05, *: p<0.05, ****: p<0.0001, Kruskal-Wallis test. This figure is a supplement to Figure 3.

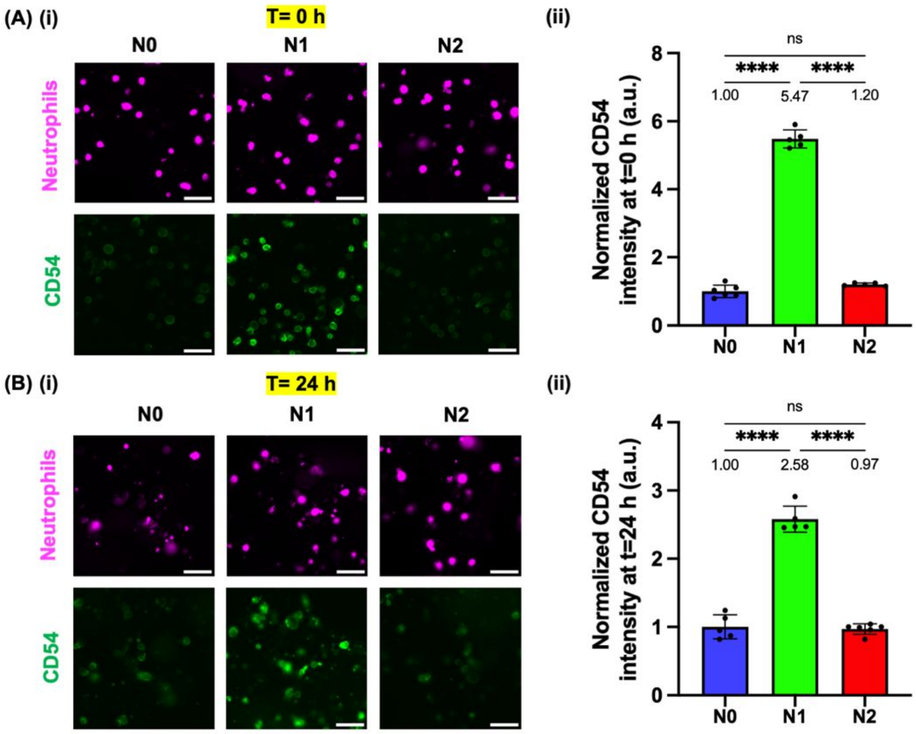


**Figure S6. N1-like and N2-like neutrophils maintain their polarization states in the NNTI-chip in scenario 2.** N1-like neutrophils expressed a significantly higher level of typical N1 marker CD54 than N2-like neutrophils throughout 24 h of on-chip culture in the presence of tumor spheroids and NK-92MI cells. **(A) (i)** Representative 20X epifluorescence images of N0, N1-like, and N2-like neutrophils (DiD, magenta) immunostained for CD54 (ICAM-1) (blue) at t=0 h after being embedded in collagen hydrogel and loaded into the NNTI-chip. Scale bar, 50 µm. **(ii)** Bar plot showing the normalized CD54 intensity of N0, N1-like, and N2-like neutrophils at t=0 h. Each data point represents the mean value of all cells per ROI. Raw values were normalized by the mean of the N0 condition. n = 5 ROIs per condition. Bars show mean ± SD with the mean values written above the points. **(B) (i)** Representative 20X epifluorescence images of N0, N1-like, and N2-like neutrophils (DiD, magenta) immunostained for CD54 (ICAM-1) (blue) at t=24 h after being embedded in collagen hydrogel and loaded into the NNTI-chip. Scale bar, 50 µm. **(ii)** Bar plot showing the normalized CD54 intensity of N0, N1-like, and N2-like neutrophils at t=24 h. One experiment was performed. ns: ≥0.05, ****: p<0.0001, ANOVA with Tukey multiple comparisons test.

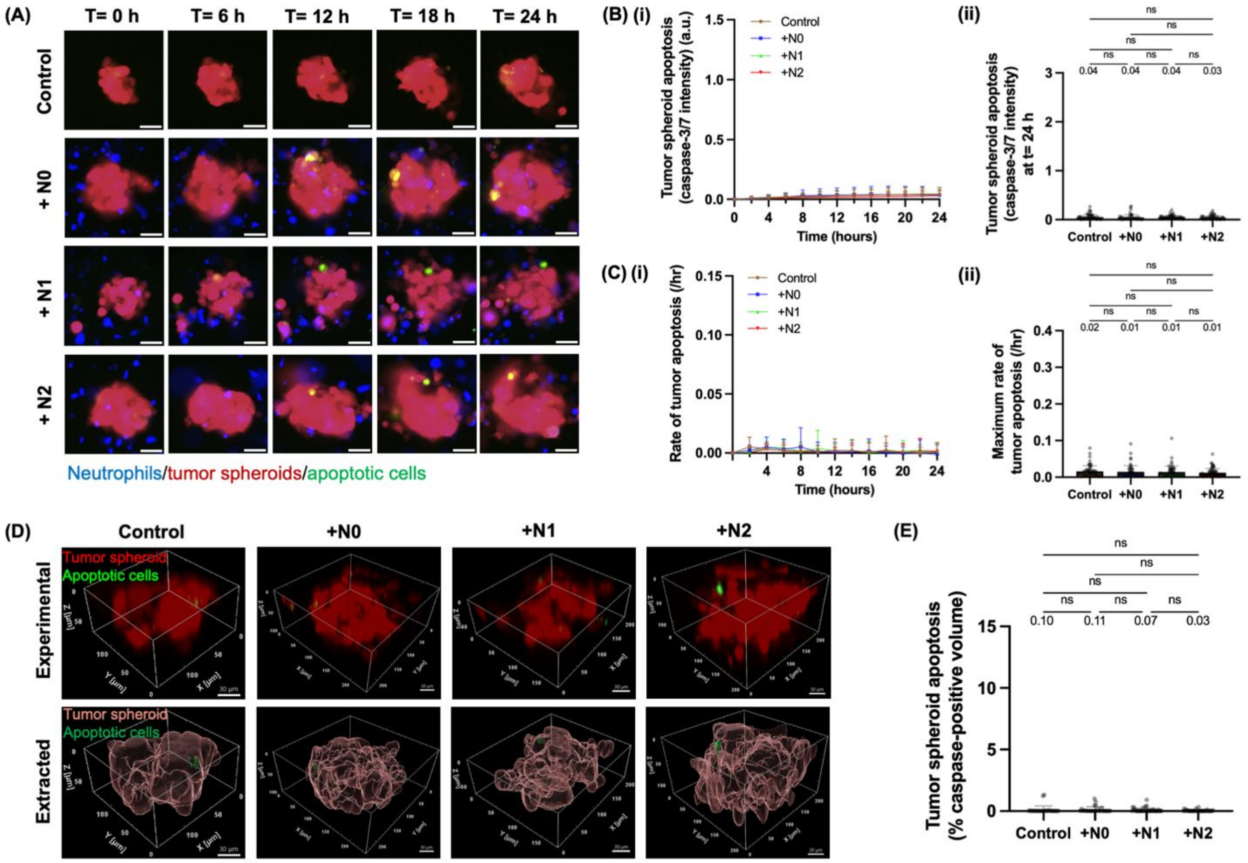


**Figure S7. N1-like and N2-like neutrophils do not affect the apoptosis of PANC-1 tumor spheroids in the absence of NK-92MI cells. (A)** Representative 10X epifluorescence images showing the apoptosis (caspase-3/7 green) of tumor spheroids (red) in the NNTI-chip at t=0 h, 6 h, 12 h, 18 h and 24 h as representative time points in the following conditions: alone (control) or co-cultured with N0, N1-like, or N2-like neutrophils (blue). Scale bar, 50 µm. **(B) (i)** Line graph showing the temporal dynamics of tumor spheroid apoptosis, quantified as normalized caspase-3/7 green intensity per spheroid, every 2 h over 24 h in all four conditions. Bars show mean + SD. **(ii)** Bar plot showing tumor spheroid apoptosis at t=24 h. Bars show mean ± SD with mean values written above the points. Each data point represents a tumor spheroid and n= 52-63 spheroids per condition. **(C) (i)** Line graph showing the rate of tumor apoptosis, defined as the increase in tumor spheroid apoptosis per hour, every 2 h over 24 h in all five conditions. Bars show mean + SD. **(ii)** Bar plot showing the maximum rate of tumor apoptosis, defined as the highest rate of tumor apoptosis at any time point over 24 h. Bars show mean ± SD. **(D)** 3D rendering of representative 10X confocal images showing the apoptosis (caspase-3/7 green) of tumor spheroids (red) fixed at t=24 h and the extraction of the apoptotic volume and the total spheroid volume by Imaris. Images were acquired as z-stacks with a 2 µm step size. Apoptotic signals co-localized with DiD-stained neutrophils were excluded to remove apoptotic neutrophils. Tumor apoptosis was quantified as the apoptotic volume divided by the total spheroid volume. Scale bar, 30 µm. **(E)** Bar plot showing the percentage of apoptotic volume per tumor spheroid. Each data point represents a spheroid and n = 29-42 spheroids per condition. Bars show mean ± SD with mean values written above the points. Two independent experiments were performed. ns: p≥0.05, Kruskal-Wallis test.

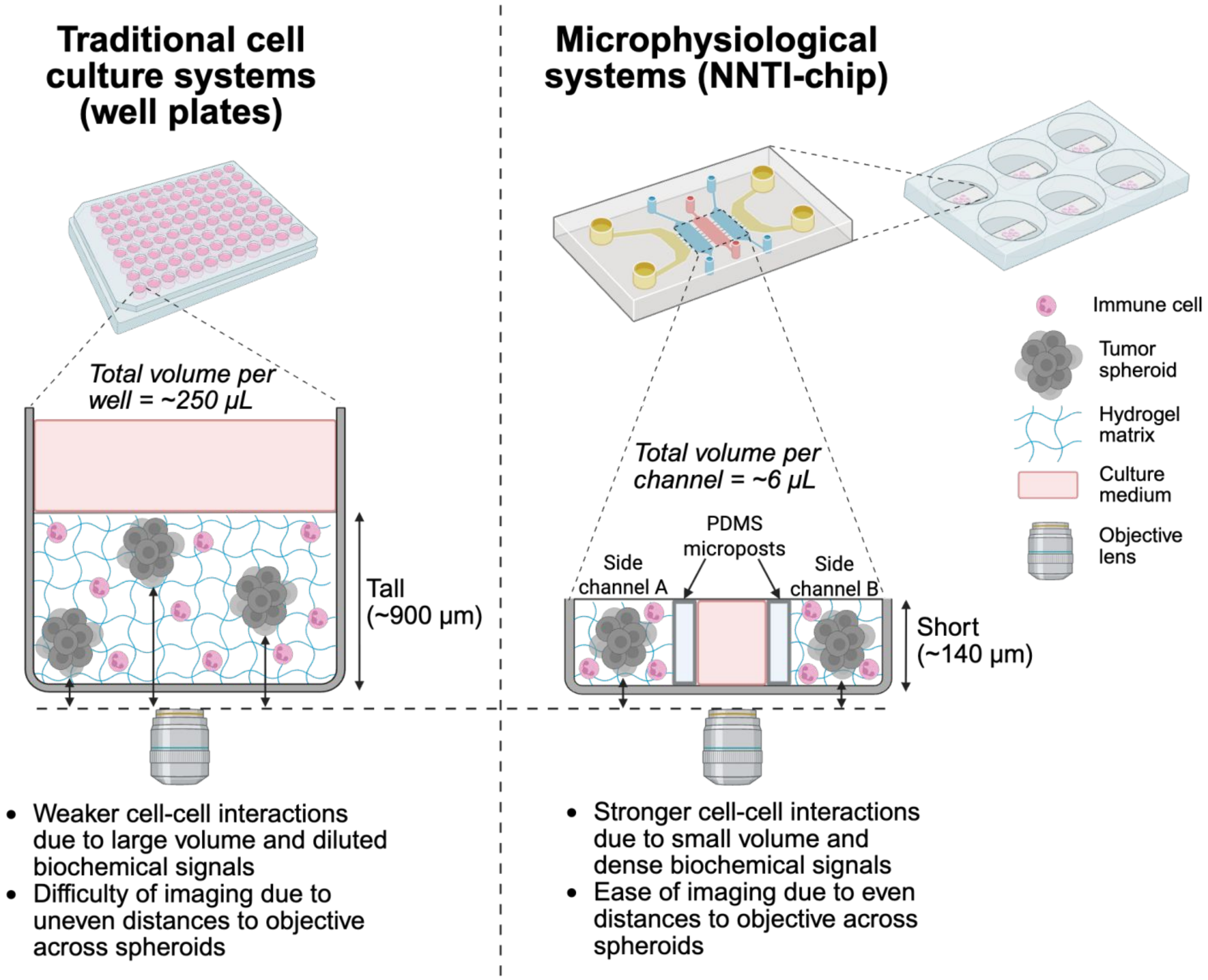


**Figure S8. Advantages of the NNTI-chip over traditional well plate-based cell culture systems for immune cell-tumor co-culture.** (1) Compared to large volumes in well plates (~250 µL for one well on a 96-well plate), the small volume of microfluidic channels in the NNTI-chip (~6 µL) also leads to faster diffusion and higher concentrations of biochemical signals and thus stronger immune cell-tumor interactions and elevated NK cell behaviors to be measured in the study. (2) In contrast to the tall thickness of the hydrogel (~900 µm) and thus uneven distances of different tumor spheroids to the objective lens of the microscope in well plates, the NNTI-chip ensures an almost even distance of different tumor spheroids to the objective lens due to the small height of its microfluidic channels (~140 µm) relative to the diameter of the spheroid (~100 µm), which makes imaging faster and easier.

## Supplementary Tables

| **NK cell behavior** | **N1 value** | **N2 value** | **Change (\|N2-N1\|)** | **Percentage change (\|N2-N1\|/N1)** |
|---|---|---|---|---|
| % migration | 12.07 | 5.11 | 6.96 | 57.7% |
| Max rate of migration | 1.31 | 0.73 | 0.58 | 44.3% |
| Speed (t=12 h) | 0.80 | 1.07 | 0.27 | 33.8% |
| Displacement (t=12 h) | 6.89 | 9.89 | 3.00 | 43.5% |
| Directionality (t=12 h) | 0.39 | 0.44 | 0.05 | 12.8% |
| Tumor apoptosis | 0.91 | 0.51 | 0.40 | 44.0% |
| Rate of tumor apoptosis | 0.15 | 0.09 | 0.06 | 40.0% |

**Table S1. The change and percentage change of the effect of N1-like and N2-like neutrophil subtypes on key NK cell behaviors measured in the NNTI-chip.** |N2-N1| represents the absolute value of the difference between the N1 condition and the N2 condition. The percentage change reflects the level of biological significance of the effect.

## Supplementary Video Legends

**Video S1**. Representative video showing the motility of NK-92MI cells (gray) after migration from the central channel into side channel A filled with neutrophils (unstained) in the NNTI-chip at t= 6 h, 12 h, 18 h, and 24 h of time-lapse imaging and the extraction of single-cell trajectories (color-coded by speed) using TrackMate (ImageJ). NK-92MI cells were tracked over 30-min intervals at each time point. Scale bar, 100 μm.

**Video S2**. Representative video showing the temporal dynamics of tumor spheroids (red) undergoing apoptosis in the presence of NK-92MI cells (blue) in the NNTI-chip over 24 h of time-lapse imaging. Apoptotic cells were stained with caspase-3/7 green. Scale bar, 100 μm.
